# Hydroxysafflor Yellow A and Anhydrosafflor Yellow B Protect Against Cerebral Ischemia/Reperfusion Injury by Attenuating Oxidative Stress and Apoptosis *via* the Silent Information Regulator 1 Signaling Pathway

**DOI:** 10.3389/fphar.2021.739864

**Published:** 2021-09-30

**Authors:** Yijia Fangma, Huifen Zhou, Chongyu Shao, Li Yu, Jiehong Yang, Haitong Wan, Yu He

**Affiliations:** ^1^ School of Pharmaceutical Sciences, Zhejiang Chinese Medical University, Hangzhou, China; ^2^ School of Life Sciences, Zhejiang Chinese Medical University, Hangzhou, China; ^3^ School of Basic Medicine Sciences, Zhejiang Chinese Medical University, Hangzhou, China

**Keywords:** hydroxysafflor yellow A, anhydrosafflor yellow B, cerebral ischemia/reperfusion injury, oxidative stress, apoptosis, SIRT1 pathway

## Abstract

Hydroxysafflor yellow A (HSYA) and anhydrosafflor yellow B (AHSYB) are the main water-soluble compounds in *Carthamus tinctorius* L. However, studies on the effect of AHSYB on cerebral ischemia/reperfusion (I/R) injury and the therapeutic effect of HSYA by regulating silent information regulator 1 (SIRT1) pathway remain obscure. In this study, we investigated whether the neuroprotective effects of HSYA and AHSYB on oxygen-glucose deprivation/reoxygenation in primary-cultured hippocampal neuronal cells and the middle cerebral artery occlusion and reperfusion model in rats are associated with the regulation of the SIRT1 pathway. *In vitro*, HSYA and AHSYB increased cell viability, depressed oxidation properties, and reduced neuronal cell apoptosis. *In vivo* results showed that HSYA and AHSYB effectively reduced infarct volume, improved neurological function, suppressed apoptosis, and decreased the oxidative stress reaction. Besides, RT-PCR and Western blot analysis showed that HSYA and AHSYB increased the mRNA and protein expressions of the main factors in the SIRT1 pathway, including SIRT1, forkhead box O (FOXO) 1, and peroxisome proliferator–activated receptor coactivator 1α (PGC1α), decreased the expression of Bax, and increased the expression of Bcl-2. The results from immunohistochemistry also showed that the expressions of SIRT1, FOXO1, and PGC1α were increased after treatment with HSYA and AHSYB. Furthermore, the neuroprotective effects of HSYA and AHSYB were abolished by EX527 (SIRT1–specific inhibitor). These results indicated that HSYA and AHSYB should be developed into potential drugs for treating cerebral I/R injury *via* the SIRT1 pathway. Although HSYA and AHSYB have different chemical structures, both of them exert similar neuroprotective properties against I/R injury *in vitro* and *in vivo*, which means that AHSYB is also a non-negligible component in safflower.

## Introduction

Currently, cerebral ischemia remains a formidable challenge in cerebrovascular disease characterized by a high rate of mortality and long-term disability around the world (Z. H. Yu et al., 2016; Zhong et al., 2020). The main treatment for cerebral ischemia is the timely restoration of the blood flow (H. Wang et al., 2019). However, reperfusion after ischemia further aggravates the brain tissue injury, which leads to a chain of pathophysiological events such as oxidative stress, apoptosis, inflammation, and even neuronal demise, namely, “cerebral ischemia/reperfusion (I/R) injury” (Deng et al., 2018; Gao et al., 2020). At present, t-PA, as a thrombolytic drug, is recommended to treat I/R injury in clinics, but this approach is limited because of its narrow therapeutic window and high risk of hemorrhagic complications (C. Y. Ma et al., 2019). Therefore, exploring new and effective drugs for alleviating cerebral I/R injury becomes an imperative task.

Oxidative stress performs an important role in the procession of cerebral I/R injury and causes strong harm to membrane lipids. Some evidence demonstrates that the neuronal membranes in the brain are vulnerable to reactive oxygenated species (ROS) at the allylic carbon of polyunsaturated fatty acids (Chioua et al., 2019). After I/R injury, excessive ROS disrupt the balance between cellular antioxidants and pro-oxidants, leading to excess production of reactive free radicals in the central nervous system (He et al., 2020). Silent information regulator 1 (SIRT1), an NAD^+^-dependent class III histone deacetylase, which first attracted interest in 1999, was used to modulate the lifespan in *Saccharomyces cerevisiae*. Since then, there has been an explosion in the research of SIRT1 in different metabolic diseases and aging processes (Khoury et al., 2018). Interestingly, in 2006, a researcher first found the neuroprotective effects of SIRT1 on hippocampal slices, and the SIRT1 signaling pathway took part in the treatment of many neurodegenerative disorders (Raval et al., 2006). Especially, in recent years, the role of the SIRT1 pathway in I/R injury has drawn our attention. For example, the expression of SIRT1 was inhibited in the middle cerebral artery occlusion and reperfusion (MCAO/R) model (M. Li et al., 2020). Forkhead box O (FOXO) and peroxisome proliferator–activated receptor coactivator 1*α* (PGC1*α*) are the direct substrates of SIRT1 (Brunet et al., 2004). By activating FOXO and PGC1*α*, SIRT1 expresses the capability to exert antiapoptosis and promote the cellular resistance activity against oxidative stress (Meng et al., 2017; Duan et al., 2019).

*Carthamus tinctorius* L., the flower of the safflower plant, also called Honghua in China, is a traditional herbal medicine widely used in treating cerebrovascular and cardiovascular diseases (L. Yu, et al., 2018). Just as the description in the *Compendium of Materia Medica*, it has potential benefits to the circulatory system with the function of “invigorating the circulation of blood” (L. Yu et al., 2020). As the representational bioactive compound extracted from safflower, hydroxysafflor yellow A (HSYA, Supplementary Figure S1A) has good therapeutic effects on stroke, such as oxygen free radical scavenging, antiapoptotic, anti-inflammatory, and antioxidative stress properties (Qi et al., 2014). In addition, anhydrosafflor yellow B (AHSYB, Supplementary Figure S1B), another water-soluble component in safflower, whose content was reported to be half as much as HSYA, also exhibited excellent antioxidative effects (Yue et al., 2015). However, previous reports mainly focus on the neuroprotective effect of HSYA and few articles mentioning about the pharmacological actions of AHSYB in cerebral I/R injury.

The hippocampus is the most sensitive area to hypoxia in the brain tissue, and hence, it is always an important objective for research studies concerning stroke. As a new target to study cerebral ischemia injury, SIRT1 is widely distributed in the hippocampus and involved in cellular oxidative stress and apoptosis. In previous studies, HSYA exerted an antiapoptotic effect in MCAO–induced rats (Deng et al., 2018), and both HSYA and AHSYB had obvious antioxidant effects (P. Huang et al., 2021). Thus, it is worthy to further study the antioxidative stress and antiapoptotic effects of HSYA and AHSYB through the SIRT1 signaling pathway.

Based on this point of view, in this article, we studied the neuroprotective effects of HSYA and AHSYB on primary-cultured hippocampal neuronal cells exposed to oxygen-glucose deprivation/reoxygenation (OGD/R) injury, in rats subjected to MCAO/R injury, and whether these two compounds showed similarities or differences in treating cerebral I/R injury. Furthermore, we also explored whether HSYA and AHSYB gave full scope to anticerebral ischemia *via* activating the SIRT1 signaling pathway.

## Materials and Methods

### Reagents

HSYA (purity >98%, lot number Z01010BA13) was obtained from Shanghai Yuanye Biotechnology Co., Ltd. (Shanghai, China). AHSYB (purity >98%) was made at the Institute of Chinese Medicine Chemistry in Zhejiang Chinese Medical University (Xu et al., 2017). Nimodipine (Nim) injection was obtained from Jumpcan Pharmaceutical Group Co., Ltd. (Jiangsu, China). The antibodies used in this study were anti-SIRT1 (orb19330, Biorbyt), rabbit anti-FOXO1 (ab70382, Abcam), rabbit anti-PGC1α (ab191838, Abcam), rabbit anti-Bax (ab32503, Abcam), rabbit anti-Bcl-2 (ab194583, Abcam), rabbit anti-GAPDH (ab181602, Abcam), goat anti-mouse IgG (H + L) secondary antibody (31,160, Thermo), and goat anti-rabbit IgG (H + L) secondary antibody (31,210, Thermo).

### Hippocampal Neuronal Culture

Primary hippocampal neuronal cells were obtained from neonatal SD rats, and the culture method was based on Yu’s previous description with some slight modifications (L. Yu et al., 2018). In brief, the hippocampus was separated from the brains and minced into small pieces and then dissociated with 0.25% trypsin-EDTA for 10 min at 37°C. Following the termination of digestion, the cell suspension was centrifuged at 1,000 rpm for 6 min and resuspended in Dulbecco’s modified Eagle medium with Ham’s F12 medium (DMEM/F12) which contained 10% fetal bovine serum (FBS). The cell suspension was plated onto 0.1 mg/ml poly-l-lysine (P-1399; Sigma)–coated 6-well plates or 96-well plates at a density of 2 × 10^5^/ml. After incubation at 37°C for 4.5 h, the medium was removed and completely replaced with the neuronal culture medium containing Neurobasal-A medium (10,888–022; Gibco) supplemented with 2% B27 (17,504–044; Gibco) and 1% glutamine. After that, half of the medium was changed every 2 days. The cells were maintained in an incubator with 5% CO_2_ at 37°C for 7 days for the following experiments.

### The Oxygen-Glucose Deprivation/Reperfusion Model and Group Design

After 7 days, the medium was removed, and the cells were washed with phosphate-buffered saline (PBS) three times. Then, the primary hippocampal neurons were incubated in a three-gas incubator (94% N_2_, 5% CO_2_, and 1% O_2_) with glucose-free Eagle’s medium at 37°C for 6 h to mimic the oxygen-glucose deprivation injury. Thereafter, the cells were incubated with a normal medium under normoxic conditions for 24 h to ensure reoxygenation. The primary-cultured hippocampal neuronal cells were divided into 11 groups: control group; OGD/R group; Nim (10 μM) group; HSYA (40 μM) group; HSYA (60 μM) group; HSYA (80 μM) group; AHSYB (40 μM) group; AHSYB (60 μM) group; AHSYB (80 μM) group; and HSYA/AHSYB + EX527 (10 μM) groups. EX527, a SIRT1–specific inhibitor, was purchased from APE × BIO Technology (Houston, United States). The control group was cultured under normal conditions. All drugs were dissolved in the culture medium and applied to the cell during the period of OGD/R injury. Also, we pretreated hippocampal cells with EX527 for 1 h before initiating HSYA or AHSYB treatment.

### Cell Viability and Lactate Dehydrogenase Release Assay

After exposure to OGD/R and different treatments, cell viability was evaluated using the cell counting kit-8 (CCK-8) assay (Beijing Zoman Biotechnology Co., Ltd., Beijing, China) according to the manufacturer’s protocol. The cells seeded into 96-well plates were treated with the CCK-8 solution and cultured at 37°C for 2 h after treatment as mentioned above. Also, the optical density (OD) values were measured at a wavelength of 450 nm.

The integrity of the cell membrane and neuronal apoptosis are related to the release of lactate dehydrogenase (LDH) (Y. Yu et al., 2018). Also, the activity of LDH in the medium was measured by ELISA in accordance with the manufacturer’s instructions.

### Hoechst 33342 Staining

We used Hoechst 33342, a DNA–binding dye, to examine hippocampal cell apoptosis (Y. Liu et al., 2018). After being processed under OGD/R conditions, the cells were fixed for 10 min in 4% paraformaldehyde at room temperature and incubated with 10 μg/ml Hoechst 33342 (Solarbio, Beijing, China) at 37°C for 25 min in the dark. Then, the cells were washed three times with PBS and observed under a fluorescence microscope (Leica, Heidelberg, Germany) immediately. The cells which exhibited intense blue fluorescence and nuclear condensation were scored as apoptotic cells (Zeng et al., 2016). Also, the percentage of the apoptotic neuronal cells was calculated by the ratio of apoptotic cells to the total cells counted.

### Middle Cerebral Artery Occlusion and Reperfusion Model Establishment

Sprague–Dawley rats (250–300 g) were obtained from the Animal Experimental Center of Zhejiang Chinese Medical University (license no. SYXK 2018–0,012). These rats were housed in a climate-controlled room (humidity of 50 ± 5% and temperature of 22 ± 2°C).

The MCAO/R model was operated with minor modifications according to Zea Longa’s method (Longa et al., 1989). After fixing the rat in the supine position, the left common carotid artery (CCA), internal carotid artery (ICA), and external carotid artery (ECA) were separated and ligated. A small hole was cut in the CCA to insert a silicone-coated 4-0 nylon monofilament (diameter of 0.28 mm, Beijing Sunbio Biotech Co., Ltd., China) into the ICA to maintain a state of ischemia. After MCAO for 1 h, the monofilament was slowly pulled to perform reperfusion and then the incision was sutured. During the surgery, all rats were positioned on a heating pad to maintain the core temperature at 37 ± 0.5°C. In the sham group, the middle cerebral artery was not occluded.

Rats were divided into nine groups, including the sham group, MCAO/R group, MCAO/R + HSYA groups (2, 4, and 8 mg/kg), MCAO/R + AHSYB groups (2, 4, and 8 mg/kg), and MCAO/R + Nim group (10 mg/kg). The drugs were administered through the tail vein immediately after MCAO/R for three consecutive days. Also, the doses of HSYA and AHSYB were determined by the clinical dosage of safflower, as given in the literature (Ramagiri and Taliyan, 2016; X.; Yang et al., 2020) and preliminary experiments in our research. For the sham and MCAO/R groups, rats were given normal saline (NS) using the same procedures. The schematic diagram of the animal experiments is shown in Supplementary Figure S2.

### Neurological Function Score

A neurological deficit assessment was conducted as per Longa’s method (Longa et al., 1989) by an investigator who was blinded to the experiment design using a five-point scale as described previously. The observations were as follows : 0, no symptoms of nerve damage; 1, rats cannot extend the ischemia contralateral forelimb; 2, rats turned to the contralateral side when crawling forward; 3, rats collapsed to the opposite side at rest; and 4, rats lost consciousness and could not be revived.

### Infarct Size Measurement

On the 3rd day after MCAO/R injury, the brain tissues were separated quickly and frozen at −20°C. After 12 min, the brains were taken and sliced into 2 mm thick coronal slices quickly. Then, the slices were stained with 2% 2,3,5-triphenyl tetrazolium chloride (TTC) at 37°C for 15 min. The normal brain tissue was rose-red, whereas the infarct area remained unstained. The brain slices were photographed using a digital camera, and the images were analyzed using Image J software.

### Hematoxylin and Eosin Staining

In brief, on the 3rd day after MCAO/R, the rats were perfused with physiological saline and 4% paraformaldehyde solution and the brains were fixed in 4% paraformaldehyde (4°C). Then, the samples were embedded in paraffin and cut into 5 μm coronal sections for a series of Hematoxylin and Eosin (HE) staining.

### TUNEL Staining

The apoptotic cells in the brain tissues were detected by TUNEL staining, and the tissue sections were treated using the *In Situ* Apoptosis Detection Kit (TaKaRa DMK500). In brief, the paraffin-embedded sections of the rat brain tissue were deparaffinized and washed with PBS and permeabilized with proteinase K (20 μg/ml) for 30 min at 37°C. After that, the tissues were incubated in a TUNEL reaction mixture for 60 min. Then, the anti-FITC HRP was added into the tissue sections for 60 min at 37°C. Images were obtained using fluorescence microscopy (Olympus BX53, Olympus, Japan).

### Measure of Reactive Oxygen Species, Malondialdehyde Level, and Antioxidant Enzymes

The levels of superoxide dismutase (SOD), malondialdehyde (MDA), ROS, and glutathione peroxidase (GSH-Px) in primary-cultured hippocampal neuronal cells and the rat serum were measured by ELISA. In brief, cells were seeded in 6-well plates, and after exposure to OGD/R, the cells were harvested to a sterile container, sonicated, and centrifuged at 12,000 rpm for 10 min and then the supernatants were collected. As for rat serum samples, they were obtained from the heart and collected by centrifugation at 4,000 rpm for 15 min at 4°C. All operations were determined with the respective ELISA kit according to the manufacturer’s instructions.

### Real-Time PCR Assay of SIRT1, FOXO1, PGC1α, Bax, and Bcl-2 mRNA Levels

The total RNA of primary-cultured hippocampal cells and ipsilateral hemisphere brain tissues of rats were isolated using the TRIzol® reagent (Invitrogen). And then, the total mRNA in each group was reverse-transcribed into cDNA with SuperScript™ III First-Strand Synthesis SuperMix (Invitrogen, United States) following the manufacturer’s instructions. Real-time PCR was performed on Power SYBR® Green PCR Master Mix (Applied Biosystems, United States). Finally, the relative mRNA expression levels of SIRT1, FOXO1, PGC1α, Bax, and Bcl-2 were quantified by the CFX384 Real-time PCR system (Bio-Rad, United States) and analyzed by the 2^−ΔΔCt^ method. The primer sequences were obtained from Shanghai Sangon Biological Engineering Technology (Shanghai, China), and their sequences are listed in Table 1.

**TABLE 1:** Primer sequences used for the real-time PCR assay.

Gene	Primer sequences (5'-3')	Genbank accession
GAPDH	GAA​GGT​CGG​TGT​GAA​CGG​ATT​TG	NM_017,008.4
	CAT​GTA​GAC​CAT​GTA​GTT​GAG​GTC​A	
SIRT1	CAG​GTA​CAG​GAA​TTG​CTC​CAC​CA	XM_003,751,934
	CTG​ATC​TCC​TTG​TTC​AAG​TTC​ACA​G	
FOXO1	CCT​CTG​TGA​GCA​GCT​GCA​ATG	NM_001,191,846.2
	CAT​GTC​ACA​GTC​CAA​GCG​CTC​AA	
PGC1α	CAG​TGT​CAC​CAC​CGA​AAT​CCT​TA	NM_031,347.1
	GGA​TCT​ACT​GCC​TGG​GGA​CCT	
Bax	CCC​CAG​GAC​GCA​TCC​ACC​AA	NM_017,059
	GGG​AGT​CTG​TAT​CCA​CAT​CAG​CAA	
Bcl-2	GGC​TAC​GAG​TGG​GAT​ACT​GGA​GAT	L14680
	CTC​TCA​GGC​TGG​AAG​GAG​AAG​ATG	

### Western Blot Analysis

The total protein was extracted from primary-cultured hippocampal neuronal cells and rat brain tissues using the total-protein extraction kit with Protease Inhibitor Cocktail (Thermo Fisher, Waltham MA, United States). Protein samples containing 60 μg proteins were separated with 8–12% sodium dodecyl sulphate (SDS)-PAGE gel and then transferred onto PVDF membranes (Millipore, Billerica MA, United States). The membranes were then incubated for 1 h with 5% fat-free milk and incubated with the primary antibodies, rabbit anti-SIRT1 (1:200, Biorbyt), rabbit anti-FOXO1 (1:4,000, Abcam), rabbit anti-PGC1α (1:1,000, Abcam), rabbit anti-Bax (1:1,000, Abcam), rabbit anti-Bcl-2 (1:500, Abcam), and rabbit anti-GAPDH (1:10,000, Abcam), overnight at 4°C. Then, the membranes were incubated with appropriate secondary antibodies (1:5,000, Thermo) for 1 h at room temperature. Ultimately, the blots were visualized by ECL chemiluminescence and the protein quantification was measured using Image J software. The relative protein levels were normalized with GAPDH.

### Immunohistochemical Assessment

We took the paraffin-embedded brain tissues to carry out immunohistochemical assessment. In brief, the paraffin sections were deparaffinized and immersed in sodium citrate buffer (10 mmol/L, pH 6.0) and subjected to microwave antigen retrieval for 15 min. Subsequently, the sections were incubated in 0.3% H_2_O_2_ for 15 min and blocked with 10% serum for 15 min. The sections were treated with one of the following primary antibodies, rabbit anti-SIRT1 (1:200, Abcam), rabbit anti-FOXO1 (1:100, CST), and rabbit anti-PGC1α (1:300, Abcam), and incubated overnight at 4°C. Then, the sections were incubated with secondary antiserum for 40 min at room temperature. Finally, the sections were stained using DAB developer kits. The expressions of SIRT1, FOXO1, and PGC1α were measured by the integrated optical density (IOD) using Image-Proplus 6.0 software.

### Statistical Analysis

All results were expressed as means ± standard deviation (SD) and differences between the groups were evaluated using one-way analysis of variance (ANOVA) followed by Tukey’s multiple comparison test. Data handling and statistical processing were performed using GraphPad Prism 8.0 (GraphPad Software, San Diego, CA, United States). A value of *p* less than 0.05 was regarded as statistically significant.

## Results

### Determination of Suitable Concentrations of Hydroxysafflor Yellow A and Anhydrosafflor Yellow B

We first incubated hippocampal cells with different concentrations of HSYA and AHSYB under normal conditions to determine the optimal concentrations of HSYA and AHSYB. As shown in Supplementary Figures S3A,B, there is no significant difference between the control group and HSYA with different doses (0–320 μM). As for AHSYB, it did not affect cell viability until the concentration up to 160 μM and so 5–120 μM was selected for the following experiments. Then, hippocampal neuronal cells were subjected to OGD/R injury to study the neuroprotective properties of HSYA and AHSYB against cerebral I/R injury. As shown in Supplementary Figures S3C,D, no significant effect was observed when HSYA was given at the doses of 10 and 20 μM and AHSYB improved cell viability significantly at a concentration range of 10–120 μM. At the dose of 80 μM, both HSYA and AHSYB exhibited more remarkable effects. Thus, three concentrations of HSYA and AHSYB (40, 60, and 80 μM) were selected for subsequent experiments.

### The Effects of Hydroxysafflor Yellow A and Anhydrosafflor Yellow B on Oxygen-Glucose Deprivation/Reperfusion–Induced Hippocampal Neuron Injury

**FIGURE 1:**
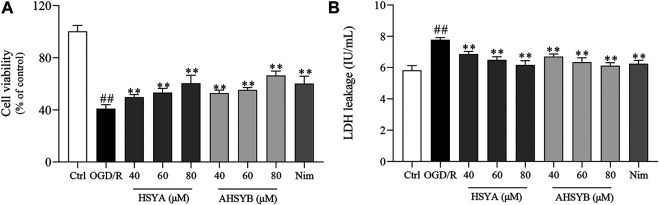
Effects of HSYA and AHSYB on cell survival and LDH release in hippocampal neurons. **(A)** Cell viability was analyzed by the CCK-8 assay. **(B)** The effects of HSYA and AHSYB on LDH leakage. Data were expressed as means ± SD; *n* = 6. ^##^ *p* < 0.01 vs. the control group; ^**^ *p* < 0.01 vs. the OGD/R group.

Cell viability and the release of LDH were examined to reflect the neuroprotective effects of HSYA and AHSYB on hippocampal cells after OGD/R. Compared to the control group, cell viability was markedly inhibited in the OGD/R group (Figure 1A). However, cell viability was increased by HSYA (40, 60, and 80 μM) and AHSYB (40, 60, and 80 μM) in a dose-dependent manner when compared with the OGD/R group. Meanwhile, the effects of HSYA and AHYSB were similar to those of the positive control drug (Nim group). In addition, once suffering from cerebral I/R injury occurs, a large amount of LDH was released to the outside of the cells. As shown in Figure 1B, after exposure to OGD/R injury, the cells released a higher amount of LDH than in the control group, indicating that the cytomembrane was broken by cerebral I/R injury. In HSYA and AHSYB treatment groups, the LDH leakage was significantly decreased showing that HSYA and AHSYB suppressed the OGD/R–induced cytotoxicity.

### The Effects of Hydroxysafflor Yellow A and Anhydrosafflor Yellow B on Amelioration of Oxygen-Glucose Deprivation/Reperfusion–Induced Oxidative Stress in Hippocampal Neurons

**FIGURE 2:**
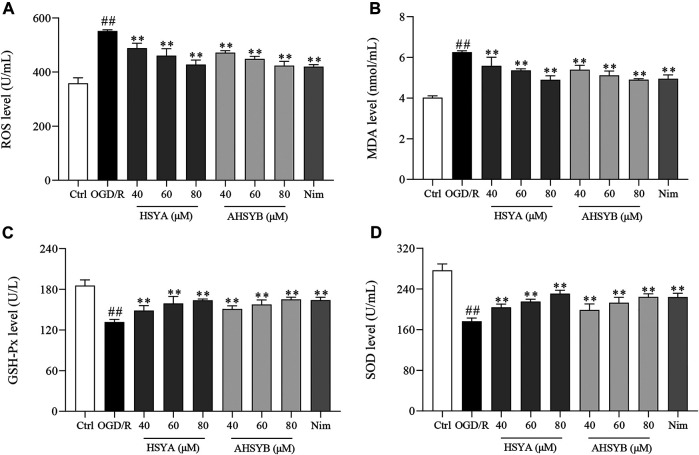
Effects of HSYA and AHSYB on ameliorated oxidative stress in OGD/R–induced hippocampal neurons. The activities of ROS **(A)**, MDA **(B)**, GSH-Px **(C)**, and SOD **(D)** caused by OGD/R induction were attenuated by HSYA and AHSYB treatment. Data were expressed as means ± SD; *n* = 6. ^##^ *p* < 0.01 vs. the control group; ^**^ *p* < 0.01 vs. the OGD/R group.

We also investigated the effects of HSYA and AHSYB on OGD/R–induced oxidative stress in hippocampal neurons by detecting the markers of oxidative stress, including ROS, MDA, SOD, and GSH-Px. Figures 2A,B show that ROS and MDA production significantly increased in the OGD/R group. Conversely, treatment with HSYA and AHSYB (40, 60, and 80 μM) decreased the levels of ROS and MDA in a dose-dependent manner. Interestingly, there was no significant difference among HSYA_80_, AHSYB_80_, and Nim groups. In addition, the activities of GSH-Px and SOD were markedly decreased in the OGD/R group relative to the control cells, whereas HSYA and AHSYB elevated the levels of GSH-Px and SOD (Figures 2C,D). In brief, these findings demonstrated that HSYA and AHSYB distinctly ameliorated OGD/R–induced oxidative stress in hippocampal neuronal cells.

### The Effects of Hydroxysafflor Yellow A and Anhydrosafflor Yellow B on Suppressed Oxygen-Glucose Deprivation/Reperfusion–Induced Apoptosis

**FIGURE 3:**
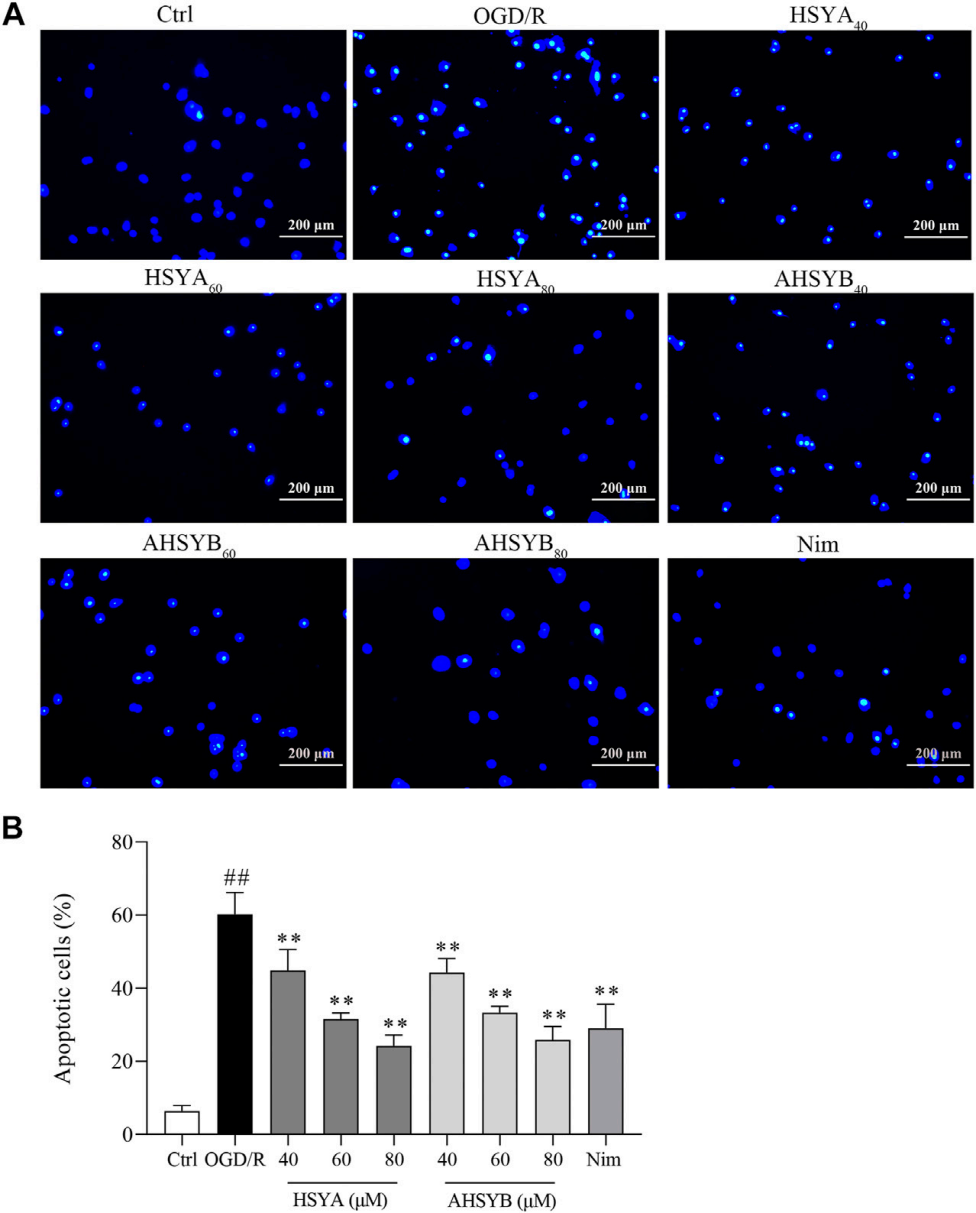
Effects of HSYA and AHSYB on cell apoptosis by Hoechst 33342 staining. **(A)** Representative images of Hoechst 33342 staining of hippocampal cells (scale bar = 200 μm, magnification = ×100). **(B)** Apoptotic cells (%). Data were expressed as means ± SD; *n* = 3. ^##^ *p* < 0.01 vs. the control group; ^**^ *p* < 0.01 vs. the OGD/R group.

Transient cerebral I/R injury leads to neuronal damage/death, and especially, the hippocampal cells in the brain are vulnerable to the attack of cerebral I/R injury. Herein, we use Hoechst 33342 to stain apoptotic nuclei to evaluate the antiapoptotic effect of HSYA and AHSYB in the hippocampus. As shown in Figure 3A, the typical morphological features of apoptosis were observed in the OGD/R group, including nuclear shrinkage, chromatin condensation, and more cells shown by bright staining. Treatment with HSYA and AHSYB significantly decreased the number of apoptotic cells characterized by less chromatin condensation. At the same time, the neuron apoptotic rate in the OGD/R group was higher than in drug-treated groups, through quantitative analysis (Figure 3B).

**FIGURE 4:**
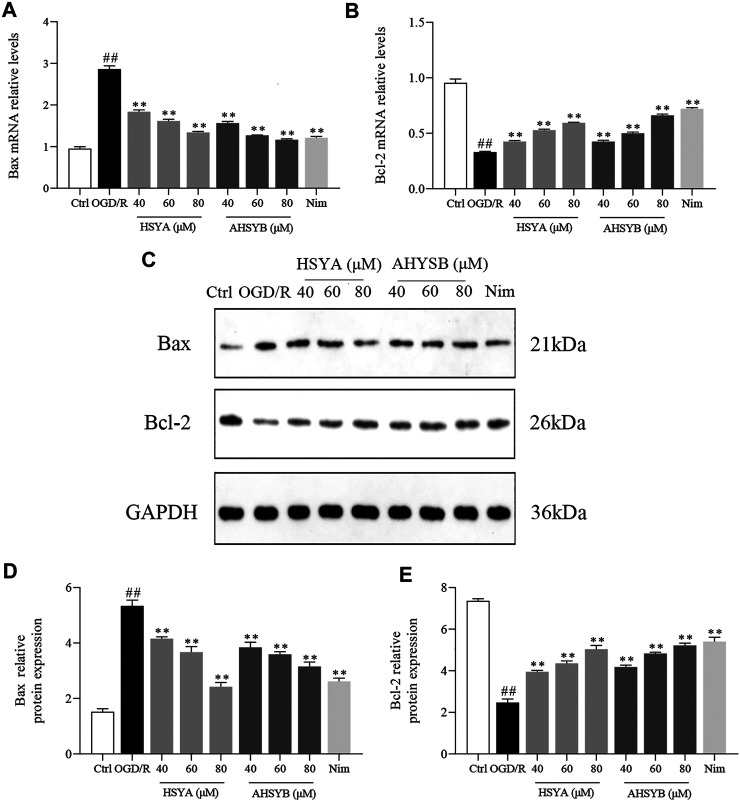
HSYA and AHSYB suppressed OGD/R–induced cell apoptosis in hippocampal neurons. The relative mRNA levels of Bax **(A)** and Bcl-2 **(B)** *in vitro.* Data were expressed as means ± SD; *n* = 5. **(C)** Representative images of Western blot for Bax, Bcl-2, and GAPDH. The quantitative analysis of the protein expressions of Bax **(D)** and Bcl-2 **(E)**. Data were expressed as means ± SD; *n* = 3. ^##^ *p* < 0.01 vs. the control group; ^**^ *p* < 0.01 vs. OGD/R group.

In addition, we also used Western blot and real-time RT-PCR to detect the expressions of apoptosis-related proteins and mRNA, including Bcl-2 and Bax. As shown in Figure 4, consistent with Hoechst 33342 staining, OGD/R induction caused significant increases in the expression of Bax, while leading to a reduction in the level of Bcl-2. Also, HSYA and AHSYB treatment changed the trends of Bax and Bcl-2. For example, HSYA and AHSYB reduced the decrease of Bcl-2 and the increase of Bax in OGD/R–induced hippocampal cells. Interestingly, HSYA and AHSYB exhibited similar pharmacodynamic action. In general, these results indicated that HSYA and AHSYB significantly inhibited the OGD/R–induced neuronal apoptosis.

### The Effects of Hydroxysafflor Yellow A and Anhydrosafflor Yellow B on the Silent Information Regulator 1 Pathway *In Vitro*

**FIGURE 5:**
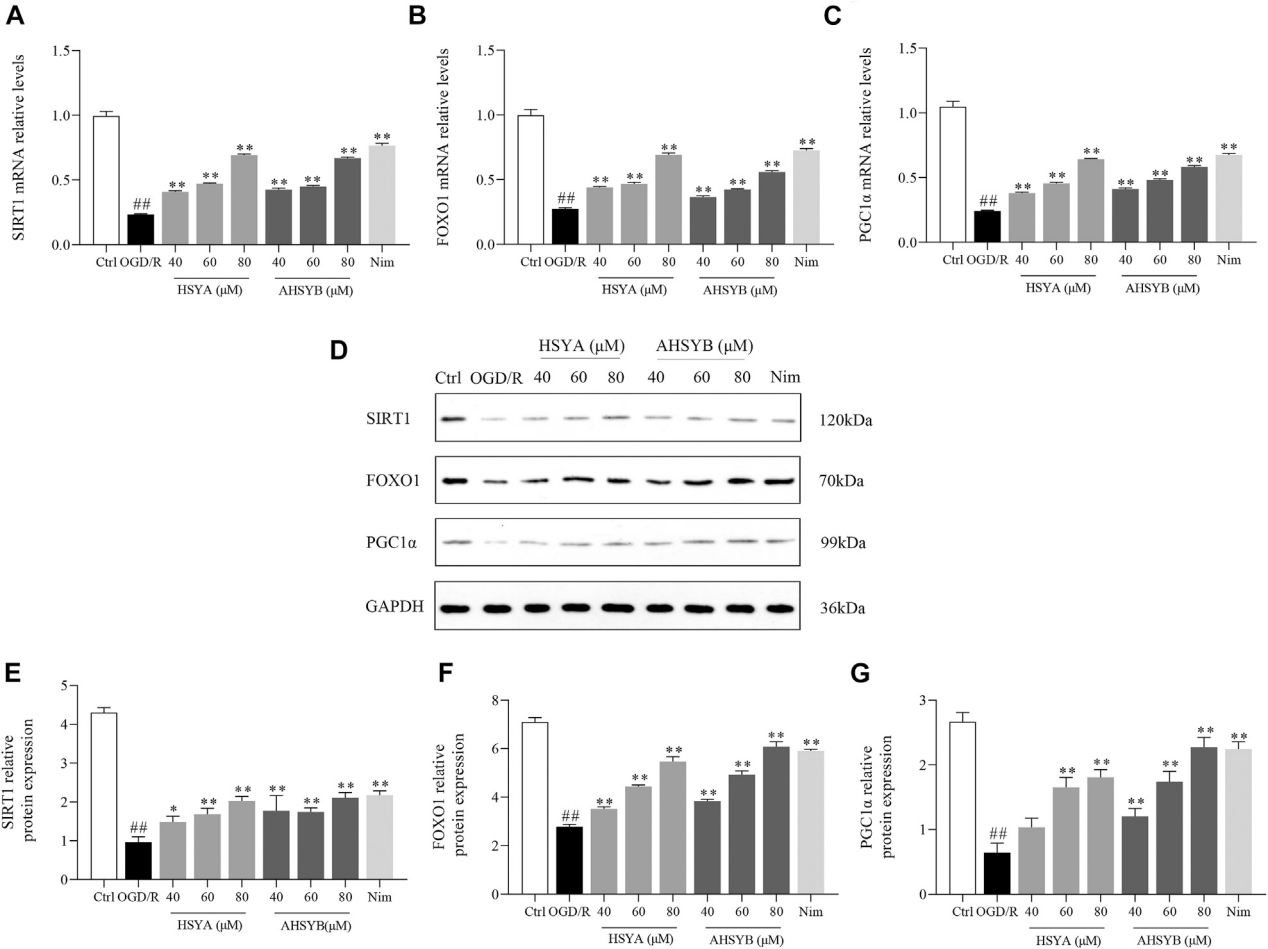
Effects of HSYA and AHSYB on the expressions of SIRT1, FOXO1, and PGC1α in hippocampal cells. The levels of SIRT1 **(A)**, FOXO1 **(B),** and PGC1α **(C)** mRNA in hippocampal cells. Data were expressed as means ± SD; *n* = 5. **(D)** Representative images of Western blot for SIRT1, FOXO1, PGC1α, and GAPDH. The quantitative analysis of the protein expressions of SIRT1 **(E)**, FOXO1 **(F),** and PGC1α **(G)**. Data were expressed as means ± SD; *n* = 3. ^##^ *p* < 0.01 vs. the control group; ^**^ *p* < 0.01 vs. the OGD/R group.

It was vital for us to understand the underlying mechanisms of HSYA and AHSYB on anticerebral I/R injury and thus RT-PCR and Western blot technology were used to explore the expressions of SIRT1, FOXO1, and PGC1α. As shown in Figures 5A–C, OGD/R exposure dramatically suppressed the mRNA expressions of SIRT1, FOXO1, and PGC1α in primary hippocampal cells. However, these inhibitions were markedly counteracted by HSYA and AHSYB treatment. In addition, Western blot technology was applied to estimate whether HSYA and AHSYB increased the expression of the SIRT1 pathway–related proteins. As shown in Figures 5D–G, the detection result was in accordance with that of the above RT-PCR and the levels of SIRT1, FOXO1, and PGC1α in the HSYA and AHSYB group were significantly increased compared with those in the OGD/R group.

### EX527 Reversed the Neuroprotective Effects Induced by Hydroxysafflor Yellow A and Anhydrosafflor Yellow B *In Vitro*

**FIGURE 6:**
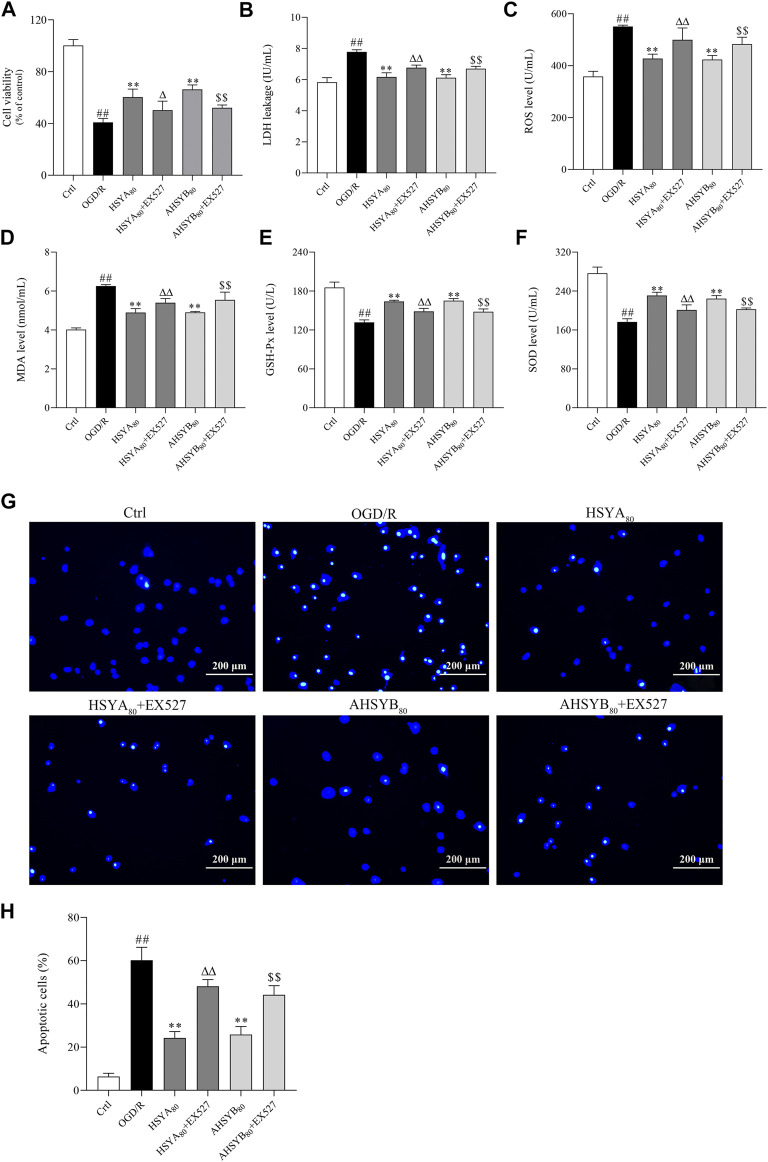
EX527 reversed the protective effects conferred by HSYA and AHSYB in primary hippocampal cells. **(A)** Cell viability. **(B)** Release of LDH. **(C)** The level of ROS. **(D)** The level of MDA. **(E)** The level of GSH-Px. **(F)** The level of SOD. Data were expressed as means ± SD; *n* = 6. **(G)** Hoechst 33342 staining (scale bar = 200 μm, magnification = ×100). **(H)** Apoptotic cells (%). Data were expressed as means ± SD; *n* = 3. ^##^ *p* < 0.01 vs. the control group; ^**^ *p* < 0.01 vs. the OGD/R group; ^Δ^ *p*<0.05 vs. HSYA_80_; ^ΔΔ^ *p*<0.01 vs. HSYA_80_; ^$$^ *p* < 0.01 vs. AHSYB_80_.

**FIGURE 7:**
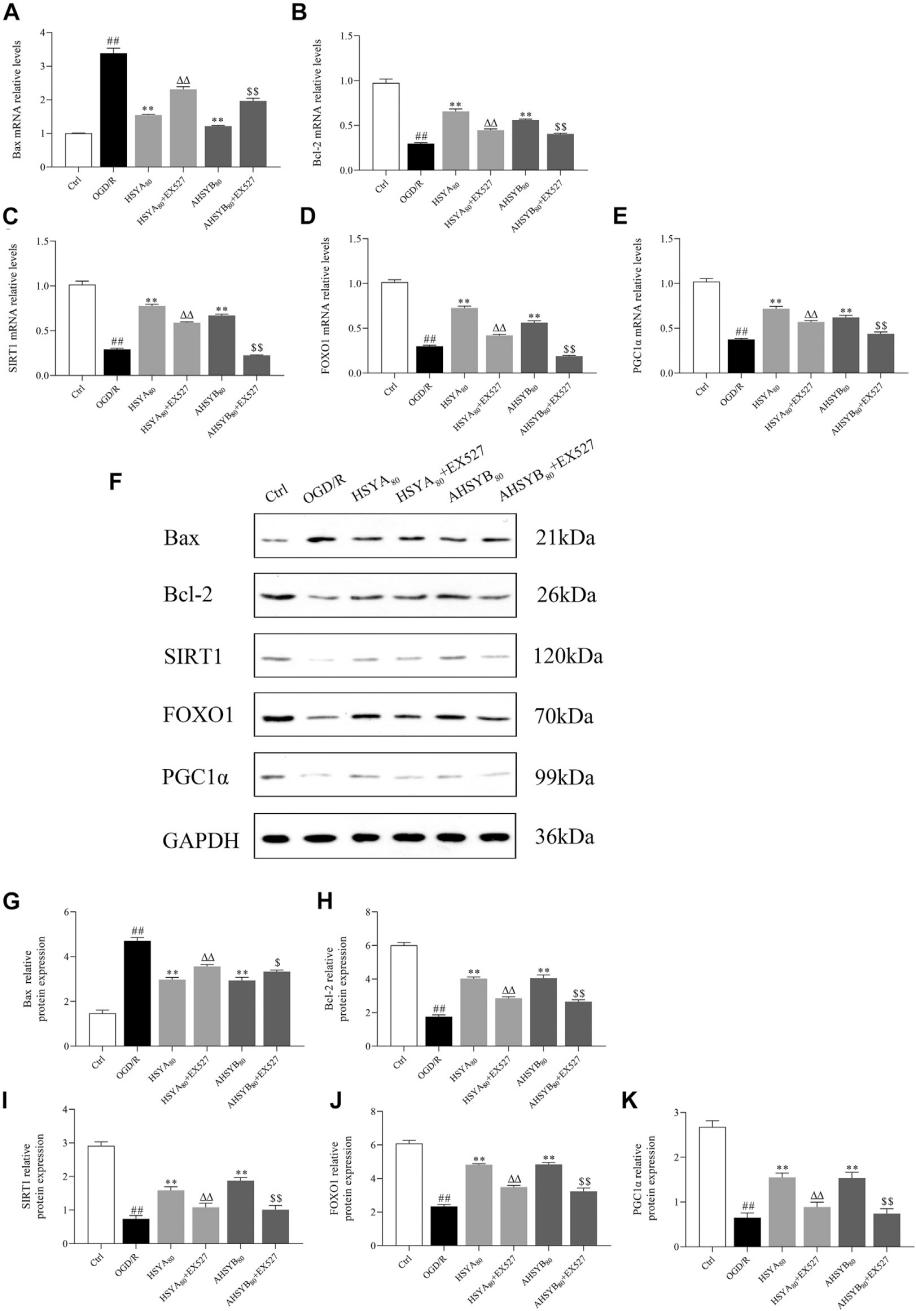
EX527 reversed the expression of protein and mRNA by HSYA and AHSYB in primary hippocampal cells. The relative mRNA expressions of Bax **(A)**, Bcl-2 **(B)**, SIRT1 **(C)**, FOXO1 **(D)**, and PGC1α **(E)**. Data were expressed as means ± SD, *n* = 5. **(F)** Representative images of Western blot for Bax, Bcl-2, SIRT1, FOXO1, PGC1α, and GAPDH. The quantitative analysis of the protein expressions of Bax **(G)**, Bcl-2 **(H)**, SIRT1 **(I)**, FOXO1 **(J)**, and PGC1α **(K)**. Data were expressed as means ± SD, *n* = 3. ^##^ *p* < 0.01 vs. the control group; **p < 0.01 vs. the OGD/R group; ^ΔΔ^ *p*<0.01 vs. HSYA_80_; ^$^ *p* < 0.05 vs. AHSYB_80_; ^$$^ *p* < 0.01 vs. AHSYB_80_.

In this part, EX527, a specific inhibitor of SIRT1, was used to inhibit the SIRT1 pathway, which further used to assess whether the neuroprotective effects of HSYA and AHSYB were dependent on the SIRT1 pathway activation. After OGD/R treatment, cells were coated with HSYA/AHSYB and EX527 and then cell viability; the release of LDH; Hoechst 33342 staining; the levels of oxidative stress markers, including ROS, MDA, SOD, and GSH-Px; and the expressions of Bax, Bcl-2, and the SIRT1 pathway–related proteins and genes were measured. As shown in Figure 6A, the SIRT1 pathway inhibitor EX527 reversed the increase in cell viability induced by HSYA and AHSYB. Also, the levels of LDH, ROS, and MDA were increased by EX527 (Figures 6B–D). GSH-Px and SOD levels were decreased by EX527 (Figures 6E,F).Similar results were shown by Hoechst 33342 staining (Figures 6G,H). Besides, Western blot analysis and RT-PCR showed that the expressions of SIRT1, FOXO1, PGC-1α, and Bcl-2 were decreased after treatment with HSYA/AHSYB and EX527 (Figure 7), which meant that the activation of the SIRT1 pathway was indeed related to the neuroprotective effects of HSYA and AHSYB.

### The Effects of Hydroxysafflor Yellow A and Anhydrosafflor Yellow B on Neurological Deficit and Infarct Volume of Middle Cerebral Artery Occlusion and Reperfusion in Rats

**FIGURE 8:**
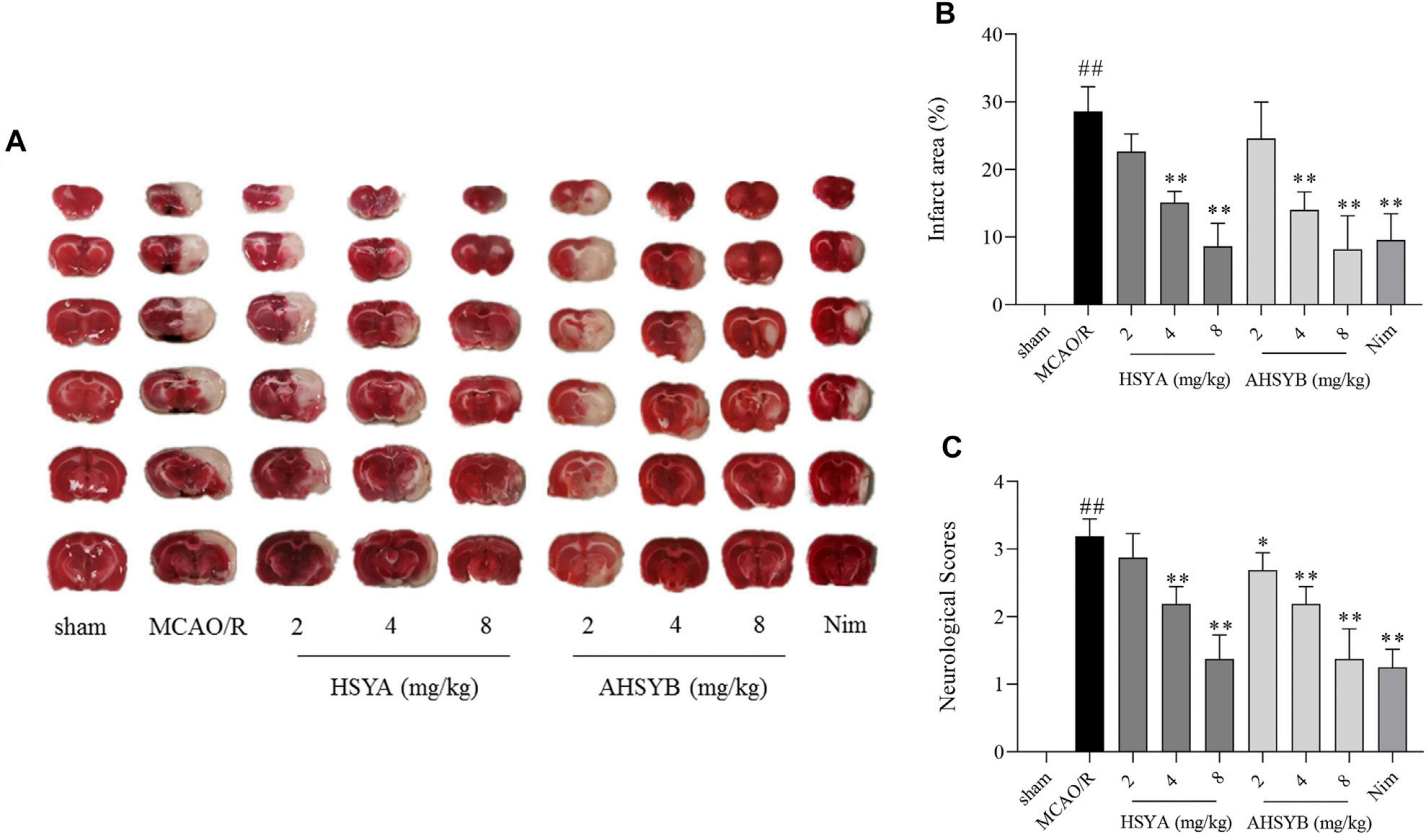
Effects of HSYA and AHSYB on neurological deficit and infarct volume. **(A)** Representative images of TTC staining. **(B)** Percentage of cerebral infarct area in the ipsilateral hemisphere; *n* = 3. **(C)** Neurological deficit scores; *n* = 8. Data were expressed as means ± SD. ^##^ *p* < 0.01 vs. the sham group; ^**^ *p* < 0.01 vs. the MCAO/R group.

The neurological deficit scores were assessed to determine the effects of HSYA and AHSYB. Also, the differences among the sham group, MCAO/R group, and treatment groups are as illustrated in Figure 8C. Rats in the MCAO/R group showed more severe neurological injuries than in the sham group. Just like the positive drug (Nim group), treatment with HSYA and AHSYB decreased the neurological deficit scores in a dose-dependent manner. Also, there were no significant differences between HSYA and AHSYB.

The extent of brain infarct volume was evaluated by TTC staining. As shown in Figures 8A,B, there was no infarction in the sham group, but the cerebral infarct area of rats in the MCAO/R group was significantly increased when compared with the sham group. Of note, treatment with HSYA, AHSYB, and Nim significantly inhibited this phenotype, which indicated that HSYA and AHSYB could protect against cerebral I/R–induced injury. The synthesis results of neurological deficit and TTC staining indicated that both HSYA and AHSYB exerted a protective effect at the dosages of 4 and 8 mg/kg and the dosage of 8 mg/kg showed more powerful effects; thus, follow-up studies were performed with this dosage.

### The Effects of Hydroxysafflor Yellow A and Anhydrosafflor Yellow B on General Histology

**FIGURE 9:**
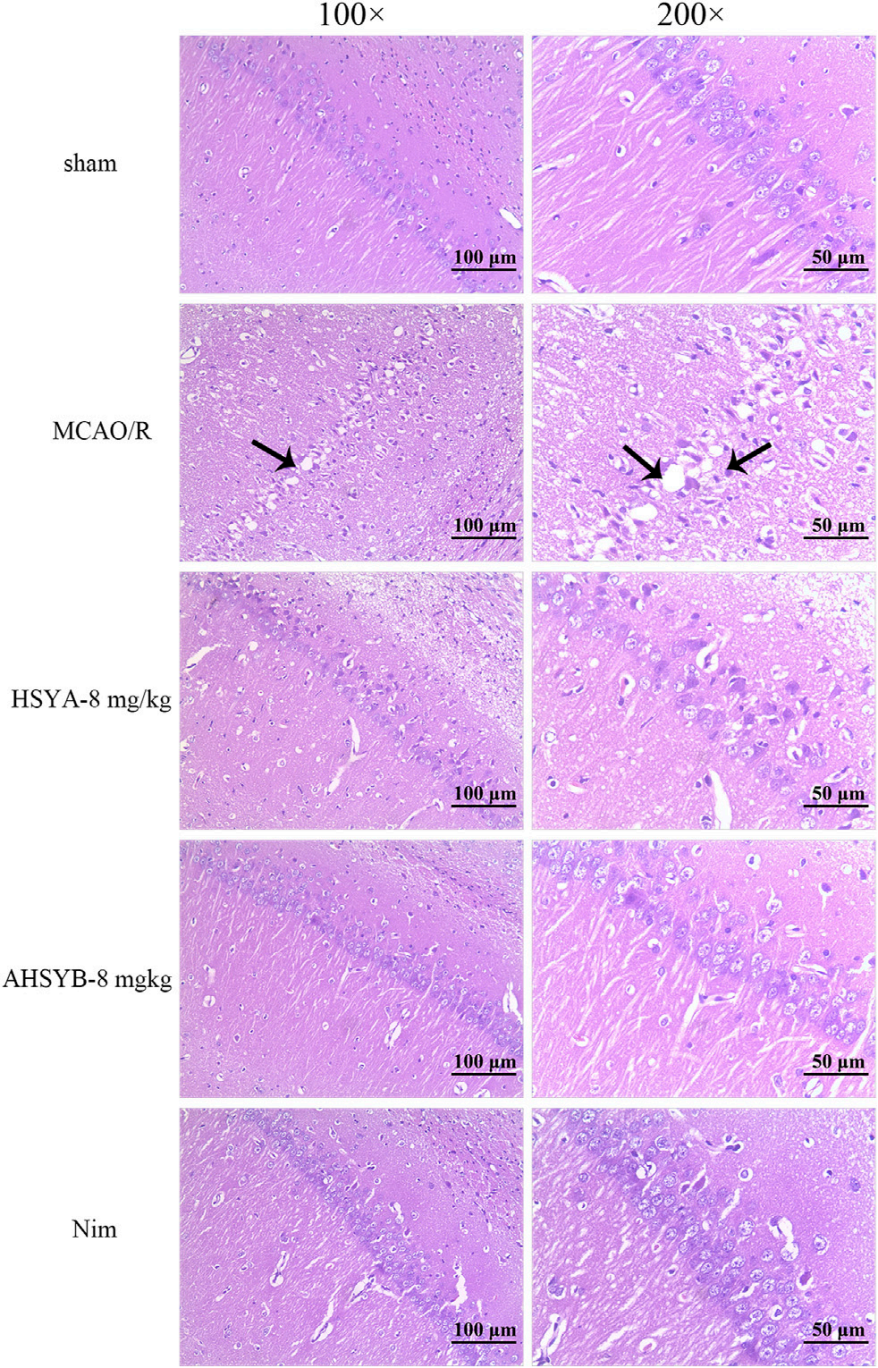
Photomicrographs of HE–stained tissue sections from the ischemic hippocampus (scale bar = 100 or 50 μm, magnification = ×100 or 200×). The necrotic cells in the MCAO/R group are shown by black tips.

We used HE staining on sections from the ischemic hippocampus to further assess the pathological damage in rats. Compared with the sham group, the structure of hippocampal neurons in the MCAO/R group was severely damaged displaying characteristics such as nuclear deformation, shrinkage, and hyperchromia. Besides, the number of cells in the MCAO/R group was decreased and they were loosely arranged. However, it is worth noting that HSYA and AHSYB treatment improved the structural damage of hippocampal neurons, which indicates that HSYA and AHSYB protected the brain from MCAO/R injury (Figure 9).

### The Effects of Hydroxysafflor Yellow A and Anhydrosafflor Yellow B on Cell Apoptosis *In Vivo*

**FIGURE 10:**
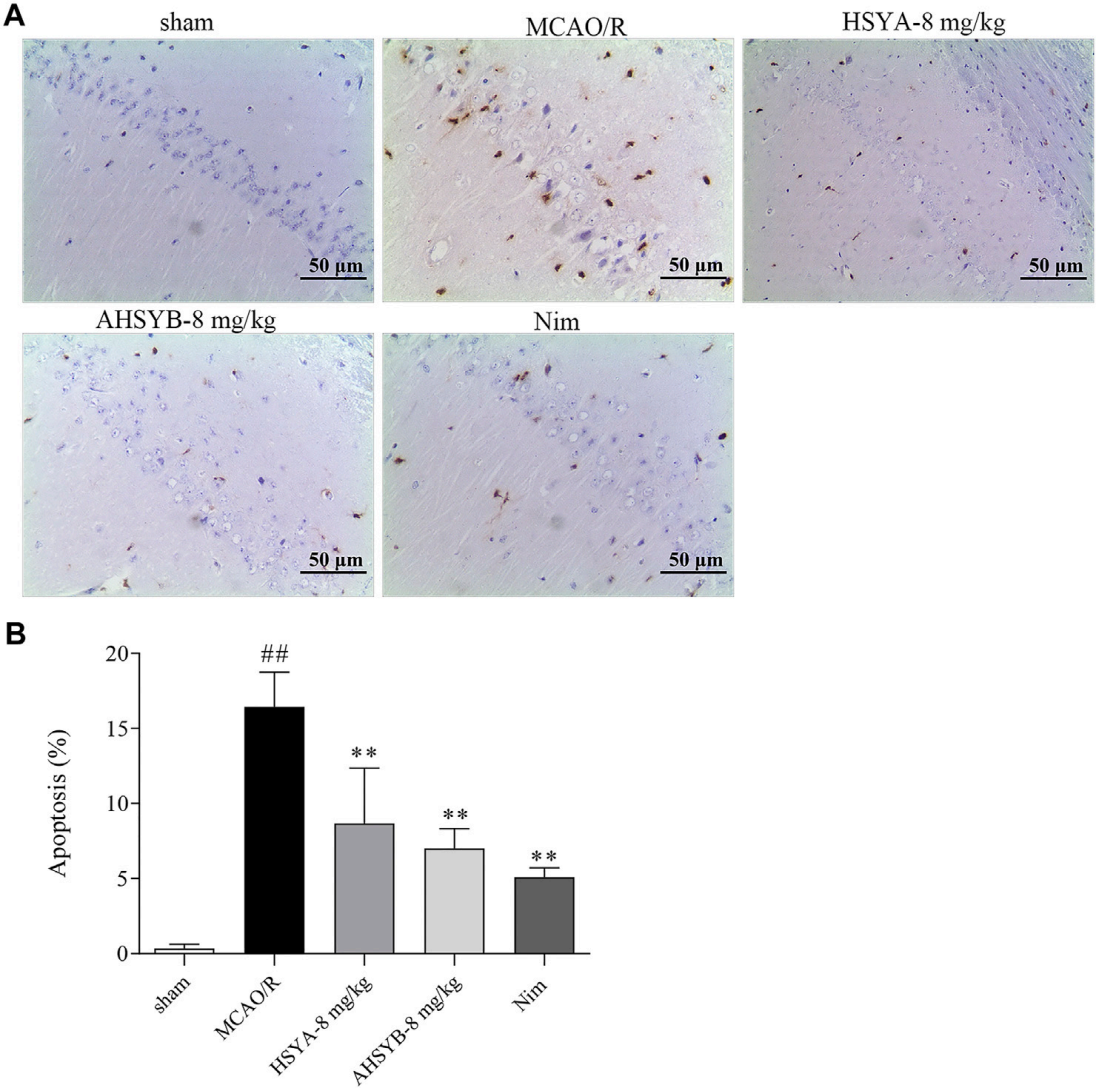
Effects of HSYA and AHSYB on cell apoptosis in the ischemic hippocampus by TUNEL staining. **(A)** Representative photomicrographs of TUNEL staining in the brain tissues (scale bar = 50 μm, magnification = ×200). **(B)** The quantitative analysis of the apoptosis rate in different groups. Data were expressed as means ± SD; *n* = 3. ^##^ *p* < 0.01 vs. the sham group; ^**^ *p* < 0.01 vs. the MCAO/R group.

**FIGURE 11:**
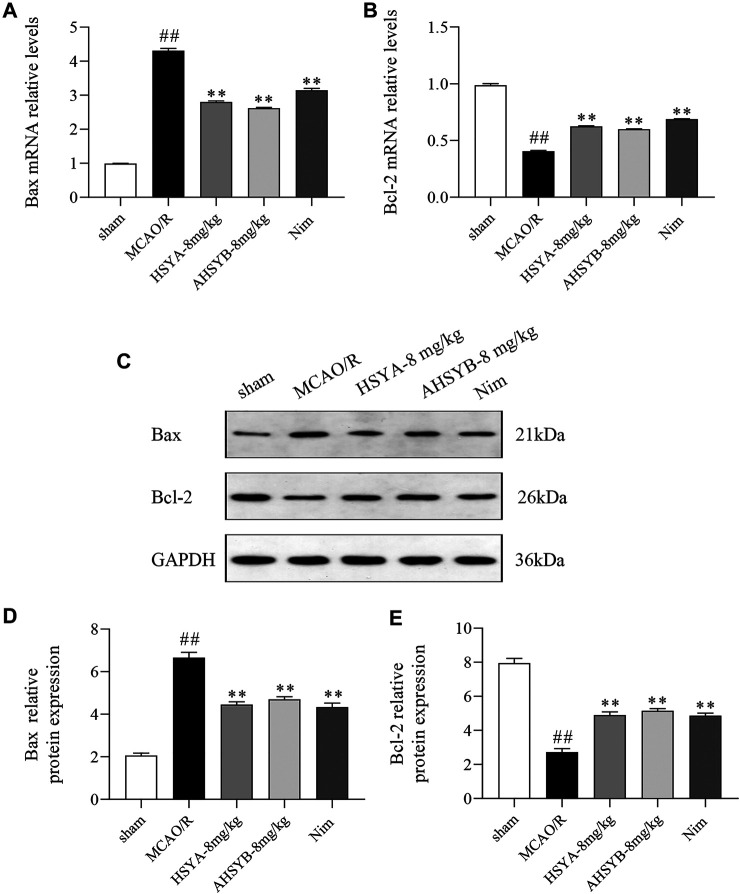
HSYA and AHSYB reduced I/R–induced apoptosis *in vivo*. The relative mRNA expressions of Bax **(A)** and Bcl-2 **(B)**. **(C)** Representative images of Western blot for Bax, Bcl-2, and GAPDH. The quantification analysis of the protein expressions of Bax **(D)** and Bcl-2 **(E)**. Data were expressed as means ± SD; *n* = 3. ^##^ *p* < 0.01 vs. the sham group; ^**^ *p* < 0.01 vs. the MCAO/R group.

The effects of HSYA and AHSYB on cell apoptosis *in vivo* were determined by TUNEL staining. As shown in Figure 10, there were nearly no apoptotic cells found in the sham group. However, a large number of apoptotic cells expressed as brown-yellow stained cells in the ischemic hippocampus of the MCAO/R group of rats were observed. Just as shown in the bar chart, the apoptosis rate in the MCAO/R group was significantly higher than in the sham group, and after HSYA and AHSYB administration, this percentage was significantly lower than in the MCAO/R group. In addition, we also detected the expressions of main apoptosis-related mRNA and proteins, including Bax and Bcl-2. According to the results shown in Figure 11 that were in line with TUNEL staining, HSYA and AHSYB reduced the increase of Bax and the decrease of Bcl-2. Meanwhile, the results were consistent with that found *in vitro*, which further confirmed that HSYA and AHSYB treatment prevented ischemia-induced neuronal apoptosis.

### The Effects of Hydroxysafflor Yellow A and Anhydrosafflor Yellow B on Oxidative Damage *In Vivo*

**FIGURE 12:**
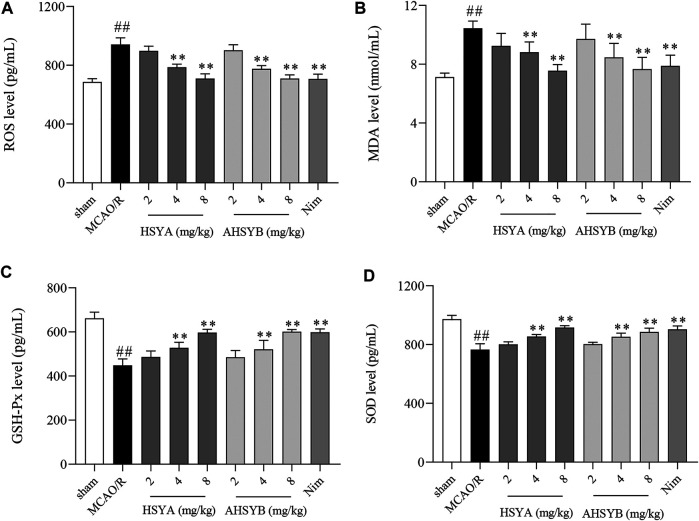
Effects of HSYA and AHSYB on oxidative stress *in vivo*. The levels of ROS **(A)**, MDA **(B)**, GSH-Px **(C)**, and SOD **(D)** in the rat serum. Data were expressed as means ± SD; *n* = 6. ^##^ *p* < 0.01 vs. the sham group; ^**^ *p* < 0.01 vs. the MCAO/R group.

The expressions of ROS, MDA, GSH-Px, and SOD in the rat serum were measured by ELISA. As shown in Figure 12, the levels of ROS and MDA were markedly increased in the MCAO/R group when compared with the sham group. However, the treatment with different doses of HSYA and AHSYB dose-dependently downregulated the amount of ROS and MDA, while GSH-Px and SOD were upregulated in drug-administered groups.

### The Effects of Hydroxysafflor Yellow A and Anhydrosafflor Yellow B on the Silent Information Regulator 1 Signal Pathway *In Vivo*

**FIGURE 13:**
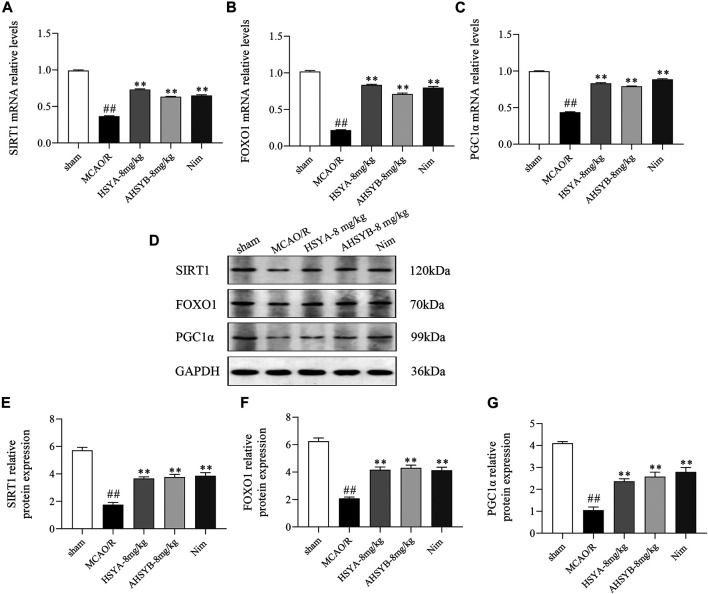
Expressions of SIRT1 **(A)**, FOXO1 **(B),** and PGC1α **(C)** mRNA in rats subjected to MCAO/R. Data were expressed as means ± SD; *n* = 3. **(D)** Representative images of Western blot for SIRT1, FOXO1, PGC1α, and GAPDH. The quantitative analysis of the protein expressions of SIRT1 **(E)**, FOXO1 **(F),** and PGC1α **(G)**. Data were expressed as means ± SD; *n* = 3. ^##^ *p* < 0.01 vs. the sham group; ^**^ *p* < 0.01 vs. the MCAO/R group.

Next, we sought to further clarify whether SIRT1 is involved in the regulation of the FOXO1 and PGC1α expression *in vivo*. As shown in Figure 13, MCAO/R induction significantly suppressed the mRNA and protein expressions of SIRT1, FOXO1, and PGC1α in ischemic brain tissues. Treatment with HSYA and AHSYB markedly increased the levels of the SIRT1 pathway–related mRNA and proteins when compared with the MCAO/R group.

**FIGURE 14:**
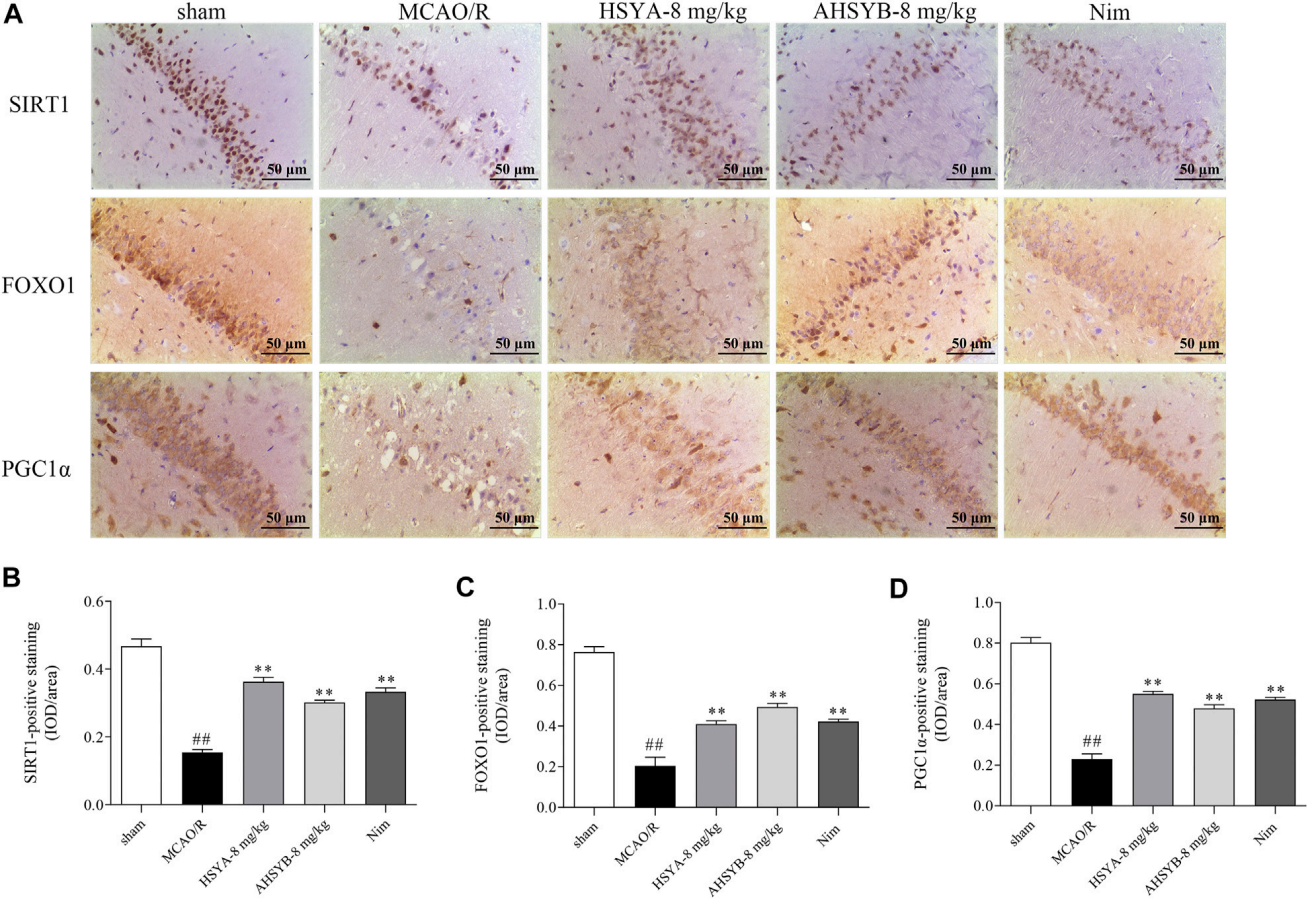
Expressions of SIRT1, FOXO1, and PGC1α in IHC. **(A)** SIRT1-, FOXO1-, and PGC1α-immunostained tissues of MCAO/R–induced rats (scale bar = 50 μm, magnification = ×200). The IHC assessments of SIRT1 **(B)**, FOXO1 **(C)**, and PGC1α **(D)** in MCAO/R–induced rats. Data were expressed as means ± SD; *n* = 3. ^##^ *p* < 0.01 vs. the sham group; ^**^ *p* < 0.01 vs. the MCAO/R group.

The results of immunohistochemical assessment in MCAO rats also revealed that SIRT1–positive staining was decreased in the brain of MCAO/R–induced rats compared with the sham group and treatment with HSYA and AHSYB dramatically increased the IOD of SIRT1. As the most important downstream targets of SIRT1, the trends of FOXO1 and PGC1α were consistent with those of SIRT1 (Figure 14). On the basis of the abovementioned results, HSYA and AHSYB exerted their anti-ischemic effect mainly through the SIRT1 pathway.

## Discussion

As we know, due to the lack of effective and applicable treatments, cerebral I/R injury is still the most frequent cause of permanent disability around the world. Although many pharmacological agents, including antiplatelet aggregation drugs, antihypertensive drugs, antioxidants, anticoagulants, and statins, have been used (C. D. Liu et al., 2021), the clinical success is limited.

HSYA, as the major component of *Carthamus tinctorius* L., is a cause for concern in recent years due to its multiple pharmacological effects in treating cerebral I/R. Hence, it was selected as an effective marker for the quality control of safflower in Chinese Pharmacopoeia (Yue et al., 2015). However, previous research studies on HSYA against I/R injury remained inadequate because of the complexity and diversity of Chinese herbal medicinal ingredients. New evidence is still essential. Intriguingly, in recent years, more and more investigators found, except for HSYA, that AHSYB is also a main component in safflower, which contains numerous hydroxyl groups and possesses antioxidative properties (Song et al., 2020). However, the underlying mechanism of AHSYB and whether it plays the same effect as HSYA are scarce.

In the present study, we revealed the beneficial effects of HSYA and AHSYB on cerebral I/R. Most interestingly, both of them showed similar pharmacological activities, including ameliorating hippocampal neuronal injury, improving neurological dysfunction, decreasing brain infarction, alleviating oxidative stress, and suppressing apoptosis. At the same time, we also found that HSYA and AHSYB displayed the neuroprotective properties against I/R injury *via* the regulation of the SIRT1 signaling pathway in the *in vitro* and *in vivo* experiments. Also, the potential mechanism is shown in Supplementary Figure S4.

In this article, primary-cultured hippocampal neuronal cells were used to determine the protective effects of HSYA and AHSYB against cerebral I/R first. The hippocampus is the most sensitive area to ischemia in the brain tissue, and especially, after 3–4 days of I/R injury, neurons in the CA1 area of the hippocampus execute apoptosis (Cho et al., 2019). By building an OGD/R–induced injury model, we discovered that both HSYA and AHSYB increased cell viability, depressed LDH leakage and oxidation properties, reduced neuronal cell apoptosis, and protected cells from OGD/R injury.

Because of the significant effects of HSYA and AHSYB on hippocampal neuronal cells, the *in vivo* anticerebral ischemia experiments were subsequently explored in rats. It is worth noting that HSYA and AHSYB also protected against I/R–induced injury, including reduced infarct volume, improved neurological function, inhibited apoptosis, and decreased oxidative stress reaction, in view of these findings.

In addition, as we know, after stroke, a series of pathological mechanisms will be produced, such as oxidative stress and apoptosis, leading to further acute injury (N. T. Ma et al., 2015). Accumulating evidence proves that oxidative stress induced by an elevated production of ROS occurs in the early stage of the cerebral ischemia cascade and has been regarded as a hallmark of the development of cerebral I/R injury. ROS damage the antioxidant defense system which leads to further cell apoptosis, dysfunction, lipid peroxidation, and even cell death. Hence, inhibition of oxidative stress helps attenuate cerebral injury (P. Li et al., 2018; Rodrigo et al., 2013). Consequently, it is necessary for us to explore if HSYA and AHSYB inhibited oxidative stress. Indeed, our data showed that after cerebral I/R injury, the level of ROS was increased and treatment with HSYA and AHSYB dose-dependently increased SOD and GSH-Px activities, which were recognized to be the vital avenue against ROS cytotoxicity (Dai, Zhang, Zhang, & Yan, 2018). Also, HSYA and AHSYB inhibited the increased levels of MDA significantly. These results suggested that the protective mechanism of HSYA and AHSYB against cerebral I/R injury was related to antioxidative stress.

It is worth noting that apoptosis is of paramount importance for cell survival in the period of cerebral I/R injury (Ren et al., 2019). Apoptosis, also called programmed cell death, caused by oxidative stress and excitotoxicity, occurs in the mammalian nervous system during development and plays a crucial role in the pathogenesis and pathological mechanism of ischemic stroke (Park, Kang, & Koh, 2020). As two common apoptotic indicators, the antiapoptotic protein (Bcl-2) and pro-apoptotic protein (Bax) have been confirmed to be closely related to neuronal apoptosis in many studies. The function of Bax is forming pores across the mitochondrial outer membrane, eventually leading to the release of cytochrome C (Y. H. Wang et al., 2018). Also, Bcl-2 acts as a mitochondrial gatekeeper, repressing cell apoptosis and counteracting the pro-apoptotic effect of Bax (P. P. Wang et al., 2020). Moreover, Bax and Bcl-2 are controlled by FOXO1 which is the direct substrate of SIRT1 to participate in the cell apoptotic process. It was reported that SIRT1 suppressed the apoptotic activities of the FOXO1 protein which directly induced the Nim gene expression and caused apoptosis in sympathetic neurons (Y. Yang et al., 2013). Furthermore, all the studies suggested that SIRT1 could activate its downstream target FOXO1 to exhibit antiapoptotic effects through apoptosis-related factors (Bax and Bcl-2) (Meng et al., 2017). Hence, in this research, we tested the expression of apoptosis-related genes and proteins (Bax and Bcl-2) to reveal the antiapoptotic effects of HSYA and AHSYB *via* the SIRT1 signaling pathway. Favorably, we found that by comparing the OGD/R or MCAO/R group and treatment with HSYA or AHSYB, the cell apoptosis rate was significantly subdued including suppressing the expression of Bax and promoting the expression of Bcl-2. In addition, in order to prove the antiapoptotic effects of HSYA and AHSYB more accurately, we carried out Hoechst 33342 staining *in vitro* and TUNEL staining *in vivo*. According to the results shown in Figure 3 and Figure 10, the hypothesis that HSYA and AHSYB regulated apoptosis to resist I/R injury was confirmed.

Indeed, the molecular mechanism of HSYA and AHSYB on I/R injury is also essential for us to elucidate. With the aim to advance this project, we executed further experiments.

SIRT1, a deacetylase widely expressed in the whole adult brain, produces a marked effect on the regulation of cell survival, energy metabolism, and antiapoptosis, whose different roles in neurological diseases have been found, indicating that it is a valuable candidate among the potential therapeutic targets in clinical practice (P. Wang et al., 2019). Activating or inhibiting the SIRT1 enzymatic activity results in a decrease or an increase in infarct volume (Zhang, Zhang, & Wu, 2018). As an example, Ding et al. pointed out that hyperbaric oxygen preconditioning increased phosphorylated neurofilament heavy polypeptides and doublecortin in the hippocampus as well as mitigated cognitive deficits in MCAO/R rats, but these effects were abolished by the SIRT1 knockdown (Ding et al., 2017). Of note, in our study, the expression of SIRT1 was decreased drastically in OGD/R–induced hippocampal cells and MCAO/R–induced brain tissues. However, HSYA and AHSYB treatment apparently upregulated the expression of SIRT1. Intriguingly, the activation effects of HSYA and AHSYB on SIRT1 were restrained when incubated with the SIRT1–specific inhibitor EX527. In view of these findings, it was indeed suggested that SIRT1 plays a major role in protection against cerebral I/R injury.

Importantly, FOXO1, one of the isoforms in the FOXO family, is closely related to regulating cell cycle, apoptosis, DNA damage repair, and oxidative stress resistance (Chae & Broxmeyer, 2011). Activating SIRT1 can promote the FOXO1 expression and transcription from the cytoplasm to the nucleus. SIRT1–mediated deacetylation has been recognized as a well-known discipline to regulate FOXO1 activity. There is a complex and interactive process between SIRT1 and FOXO1. On the one hand, SIRT1 controls the nuclear shuttling of FOXO1 and regulates FOXO1 activity, depending on the target gene or cell type (X. Z. Huang et al., 2019). On the other hand, after the deacetylation of FOXO1, SIRT1 not only reduces the level of FOXO1 but also inhibits its ability to induce cell apoptosis, ultimately protecting cells from death. In addition, the SIRT1/FOXO1 pathway is a vital signaling pathway considered to be involved in regulating oxidative stress and apoptosis. On this basis, we investigated the possible connection between these two issues. Treatment with HSYA and AHSYB led to an increase in the expression of FOXO1 and the inhibition of apoptosis-related indexes. Furthermore, the effects of HSYA and AHSYB were blocked by EX527, which meant that HSYA and AHSYB alleviated apoptosis induced by I/R injury through regulating FOXO1 *via* SIRT1.

Another target of SIRT1 is PGC1α. Several studies have identified that this target is connected with ischemic neuroprotection (Koronowski & Perez-Pinzon, 2015). PGC1α, a transcriptional coactivator, was abundantly expressed in the tissues with high metabolic rates, especially neurons. When neurons are subjected to oxidative stress, PGC1α exerts its antioxidative effect by increasing the expression of SOD (L. C. Li, Liu, Cao, He, & Bai, 2019). It also plays a pivotal role in modulating the ROS level and the expressions of various antioxidative proteins *in vivo* and *in vitro*. In addition, PGC1α is important for SIRT1 to improve stroke-related conditions. In the alpha-lipoic acid and icariin paradigms mentioned, SIRT1 catalyzed the deacetylation of PGC1α and enhanced the expression of PGC1α (Zhu et al., 2010; Fu et al., 2014). Therefore, it is reasonable for us to speculate that SIRT1 regulates PGC1α to treat I/R injury. In truth, in the current study, we illustrated that HSYA and AHSYB protected against I/R injury by upregulating the expression of PGC1α, and it indeed seems to exist as a crosstalk between SIRT1 and PGC1α, according to the results of immunohistochemistry (IHC), Western blot, and RT-PCR. Among these molecular mechanisms, it supports our current study that both HSYA and AHSYB exerted antioxidative stress and antiapoptotic effects by activating the SIRT1 signaling pathway.

## Conclusion

In summary, our results demonstrated that HSYA and AHSYB should be deemed into potential therapeutic drugs in the treatment of OGD/R–induced neuronal damage and MCAO/R–induced I/R injury by alleviating oxidative stress and apoptosis *via* the SIRT1 signaling pathway. Besides, according to our findings, there is no difference between HSYA and AHSYB in *in vitro* and *in vivo* studies. However, more in-depth studies of HSYA and AHSYB concerning stroke are needed to be investigated in the future.

## Data Availability

The original contributions presented in the study are included in the article/Supplementary Material; further inquiries can be directed to the corresponding authors.

## Abbreviations

AHSYB, anhydrosafflor yellow B; EX527, inhibitor of SIRT1; FOXO, forkhead box O; GSH-Px, glutathione peroxidase; HSYA, hydroxysafflor yellow A; I/R, ischemia/reperfusion; LDH, lactate dehydrogenase; MCAO/R, middle cerebral artery occlusion and reperfusion; MDA, malondialdehyde; Nim, nimodipine; NS, normal saline; OGD/R, oxygen-glucose deprivation/reperfusion; SIRT1, silent information regulator 1; PGC1α, peroxisome proliferator–activated receptor coactivator 1α; ROS, reactive oxygen species; SOD, superoxide dismutase; TTC, 2,3,5-triphenyl tetrazolium chloride.
