# Supplementary figures and images for: Hydroxysafflor Yellow A and Anhydrosafflor Yellow B Protect Against Cerebral Ischemia/Reperfusion Injury by Attenuating Oxidative Stress and Apoptosis via the Silent Information Regulator 1 Signaling Pathway
Source: Front Pharmacol. 2021 Sep 30;12:739864. doi: 10.3389/fphar.2021.739864 (PMC8514692; doi:10.3389/fphar.2021.739864)

**Images of the original western blots**

Fig.1


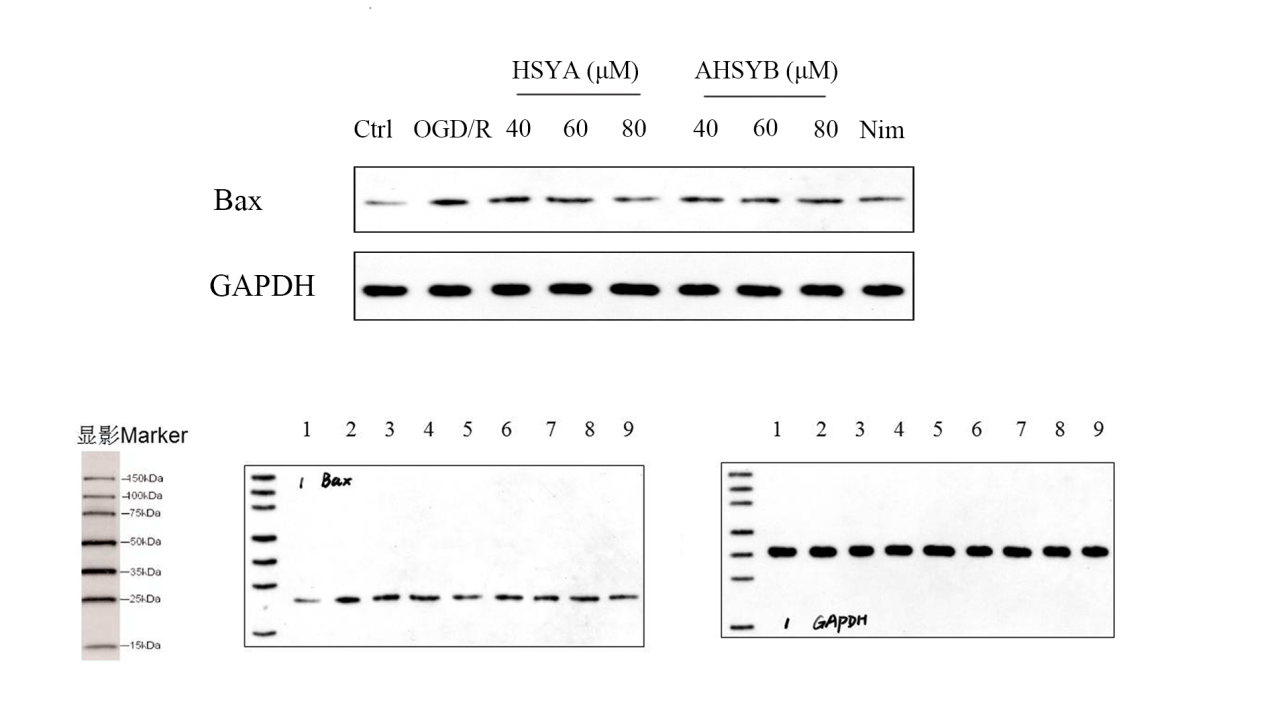


Fig.2


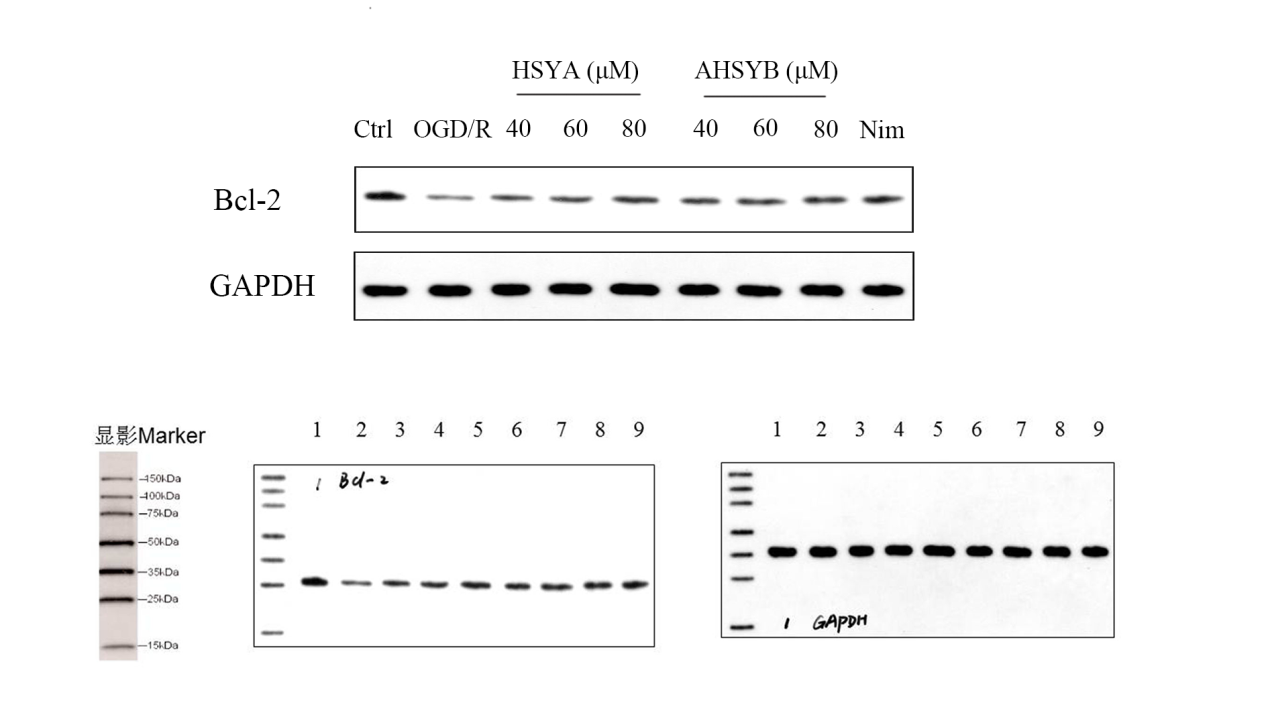


Fig.3


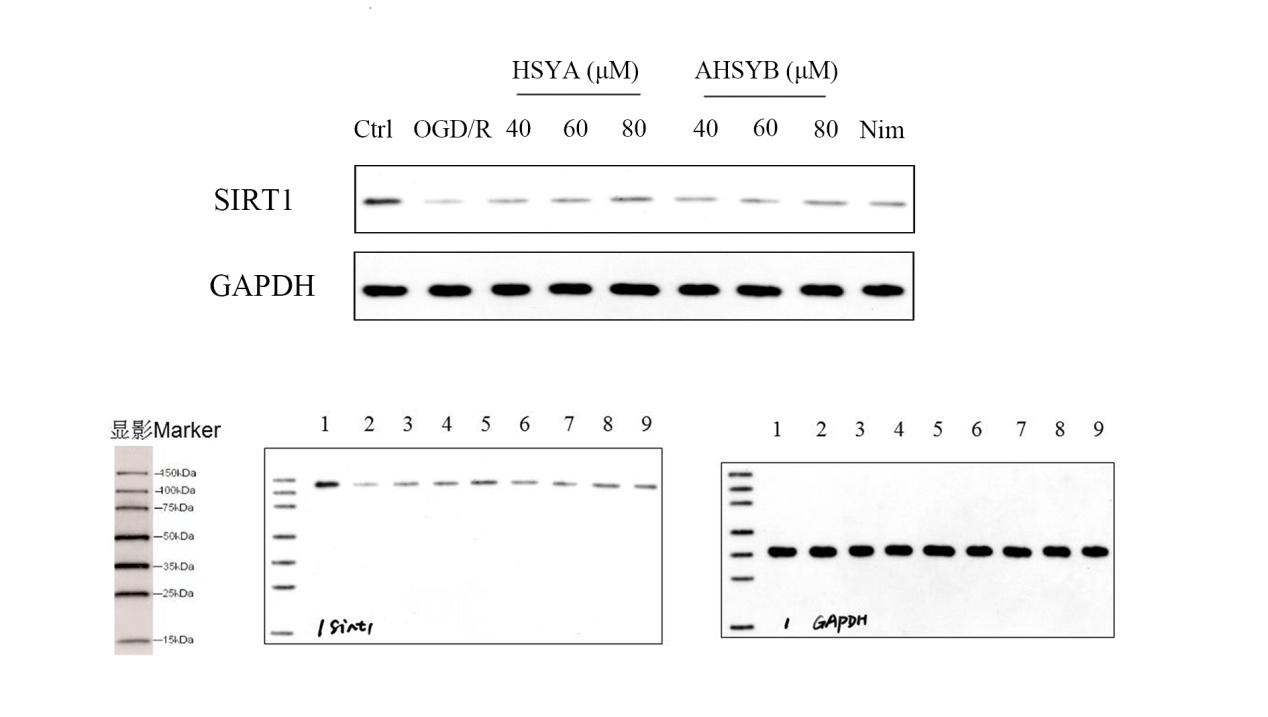


Fig.4


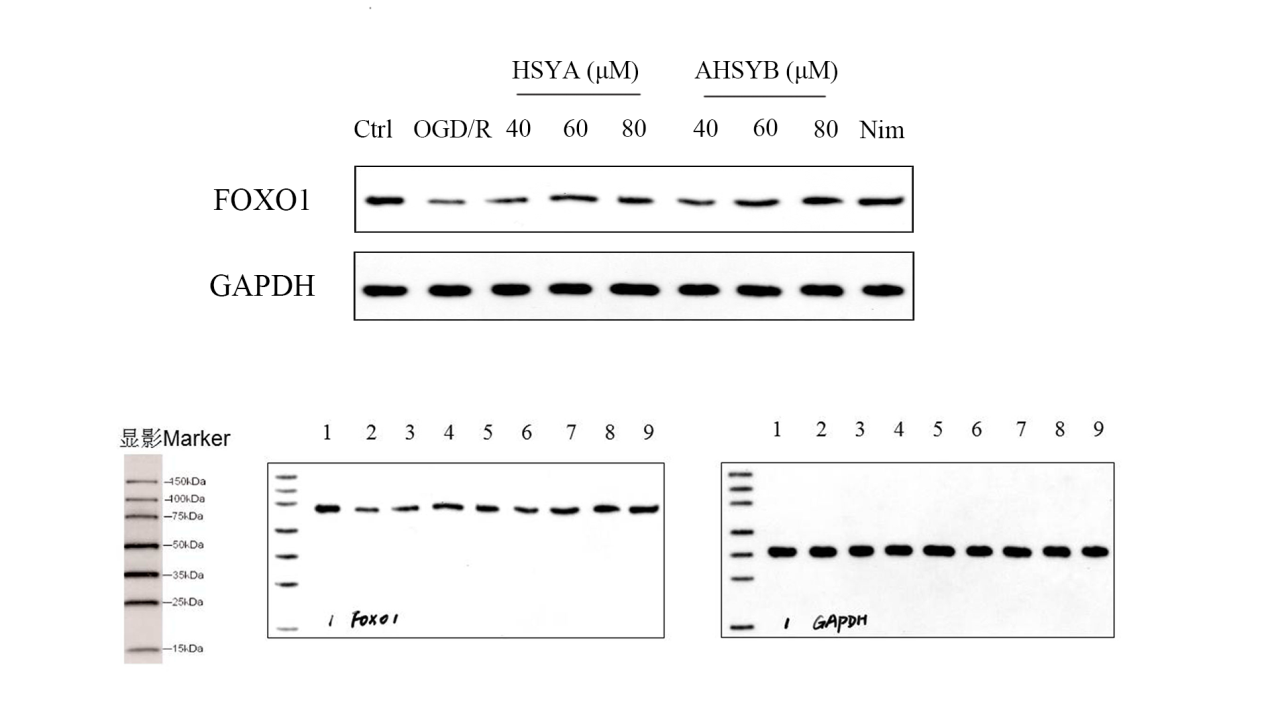


Fig.5


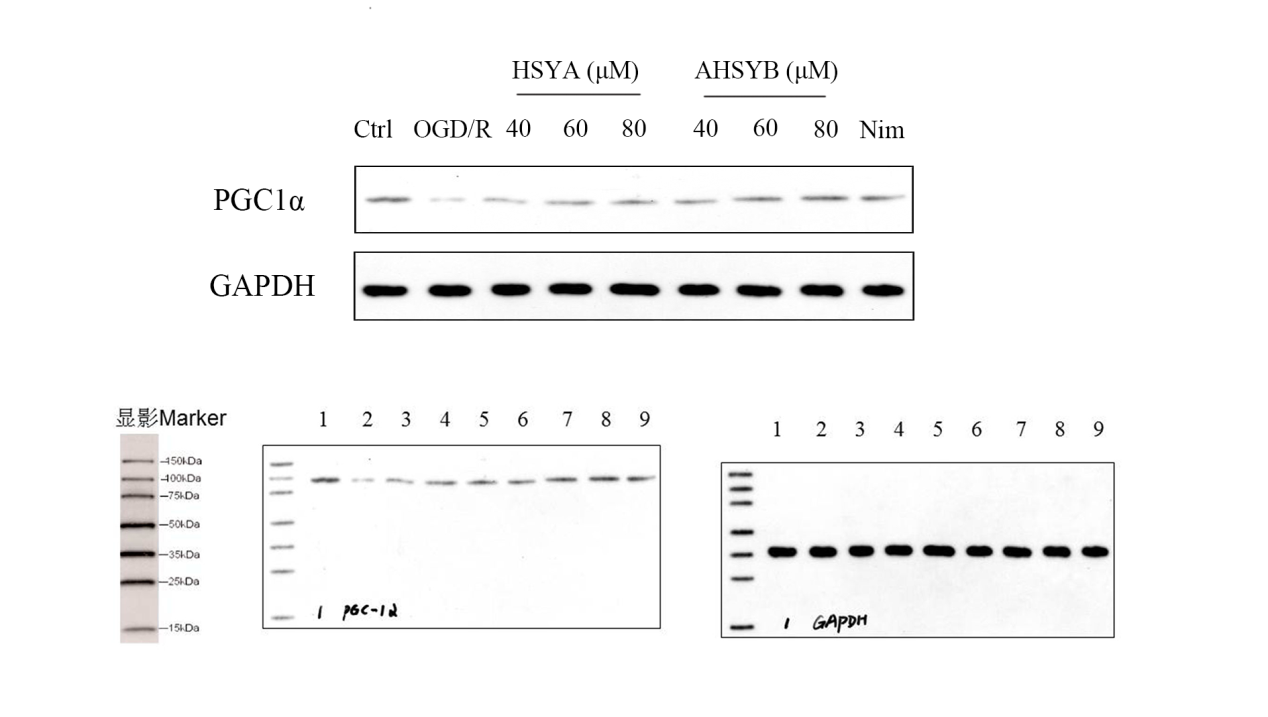


Fig.6


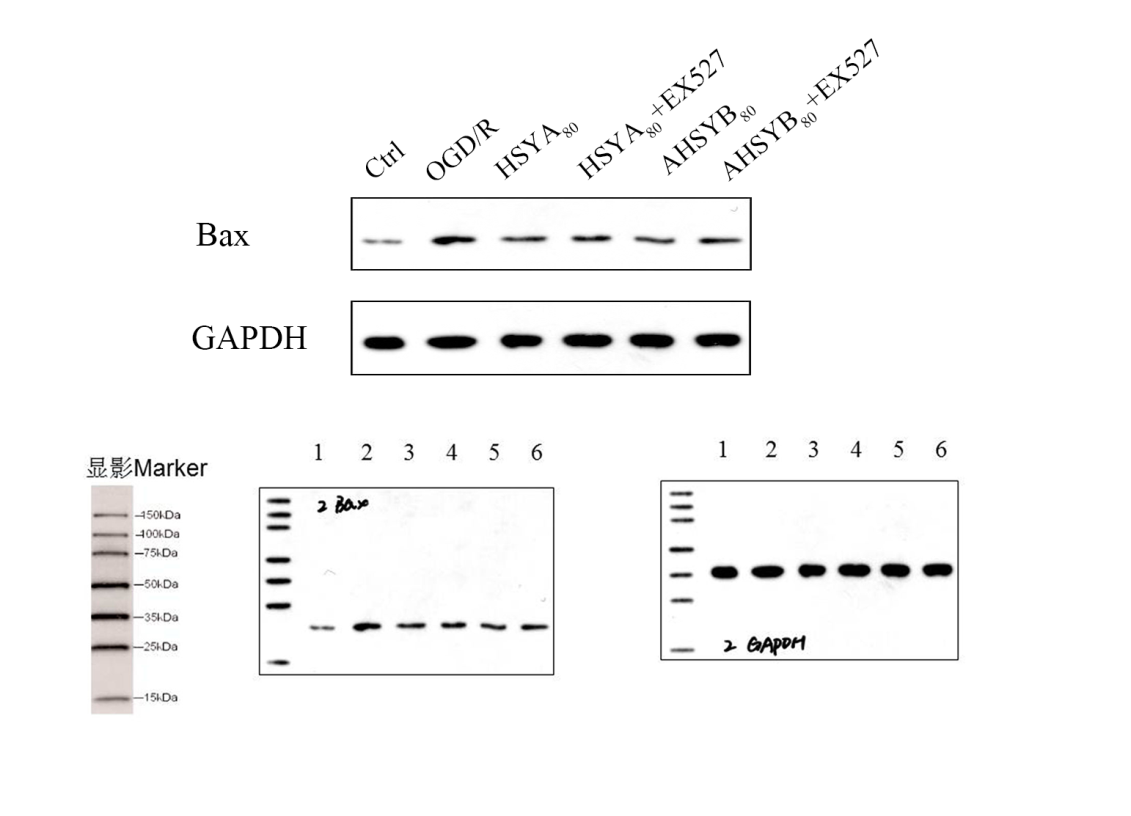


Fig.7


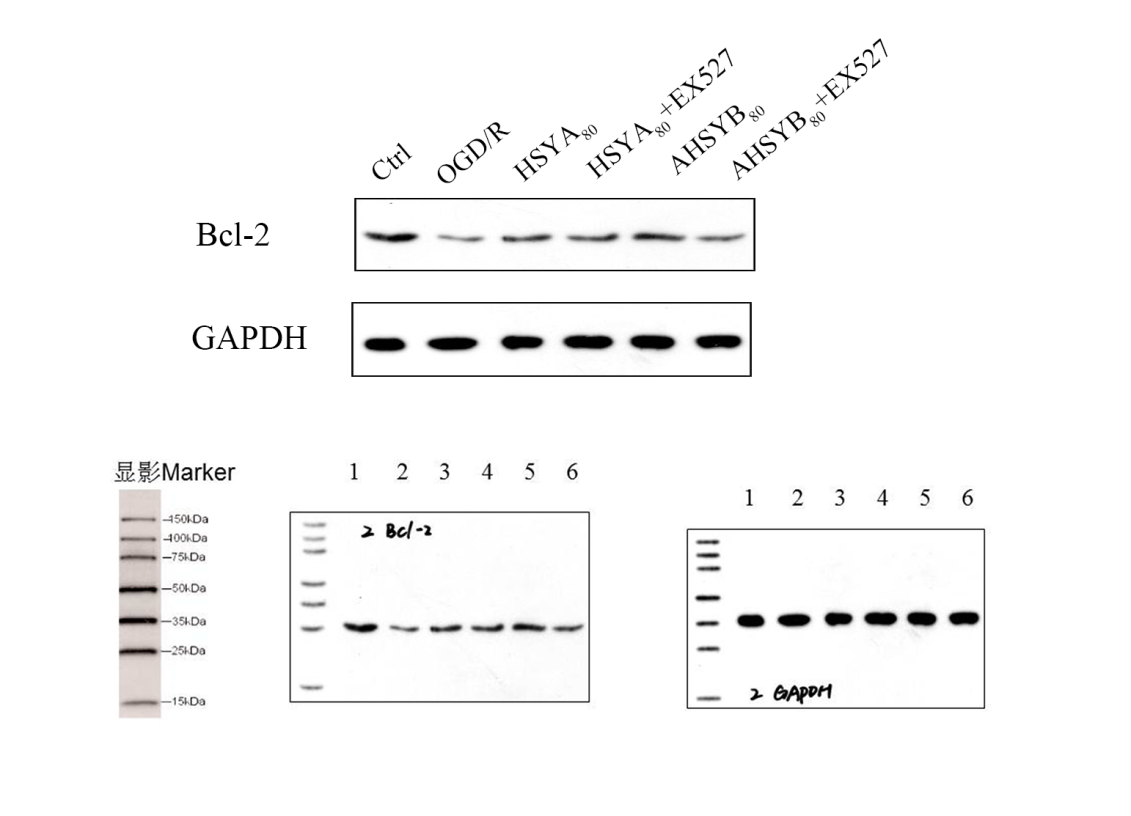


Fig.8


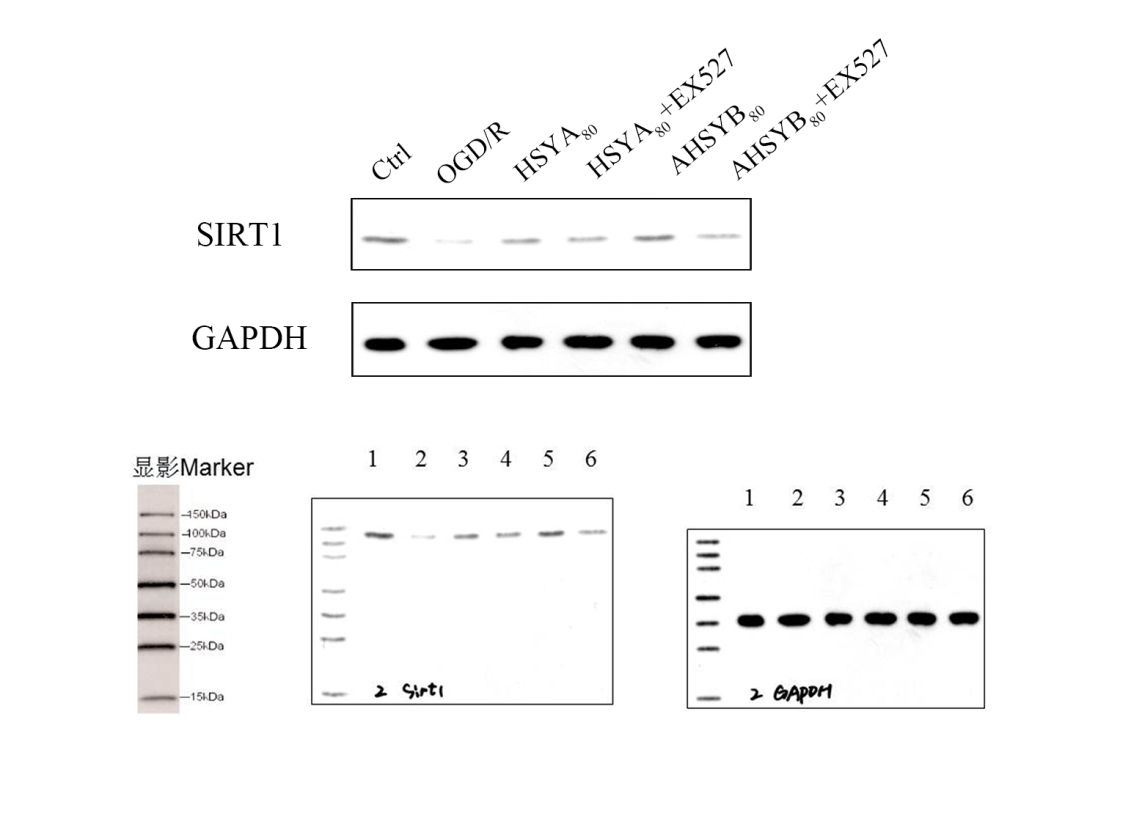


Fig.9


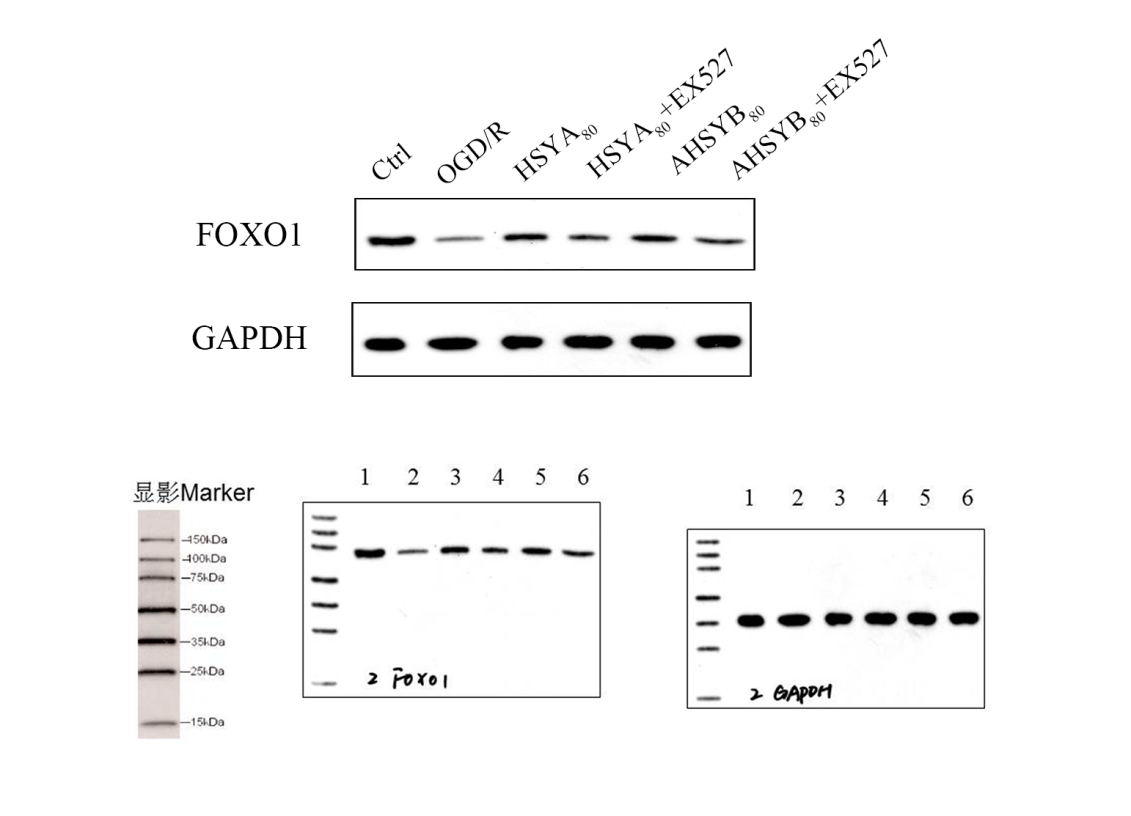


Fig.10


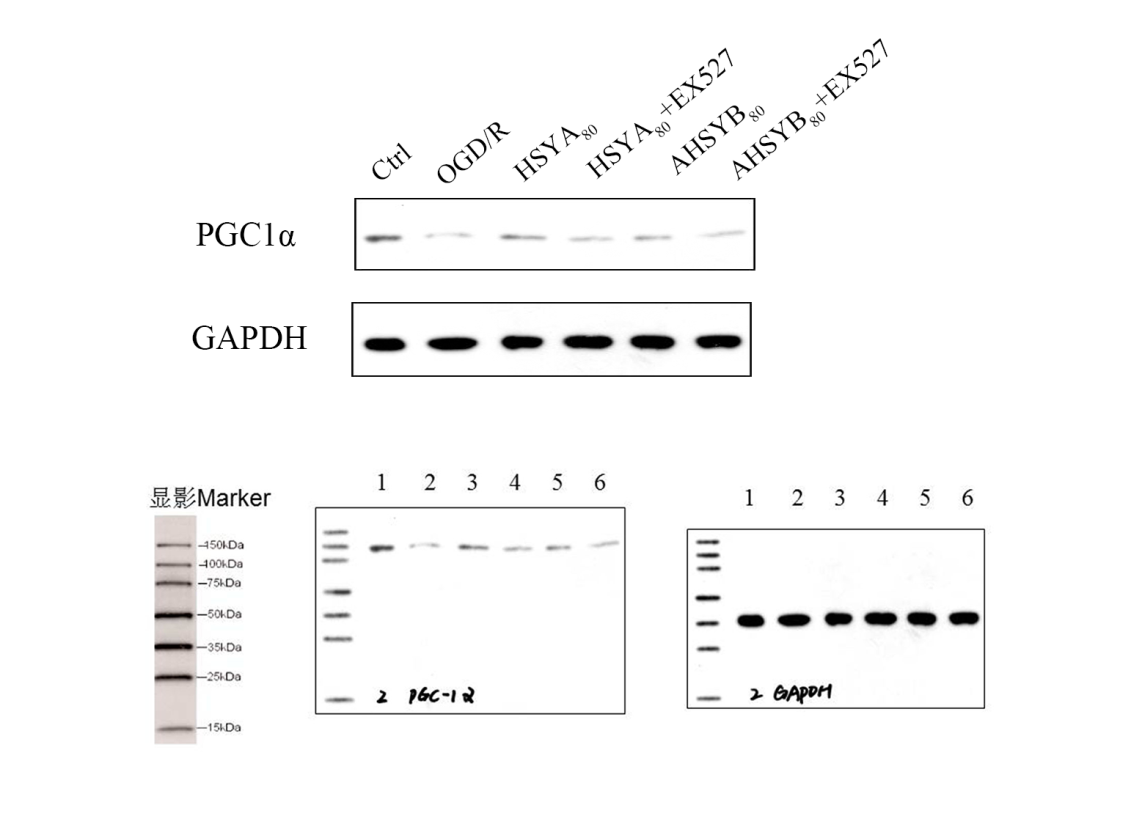


Fig.11


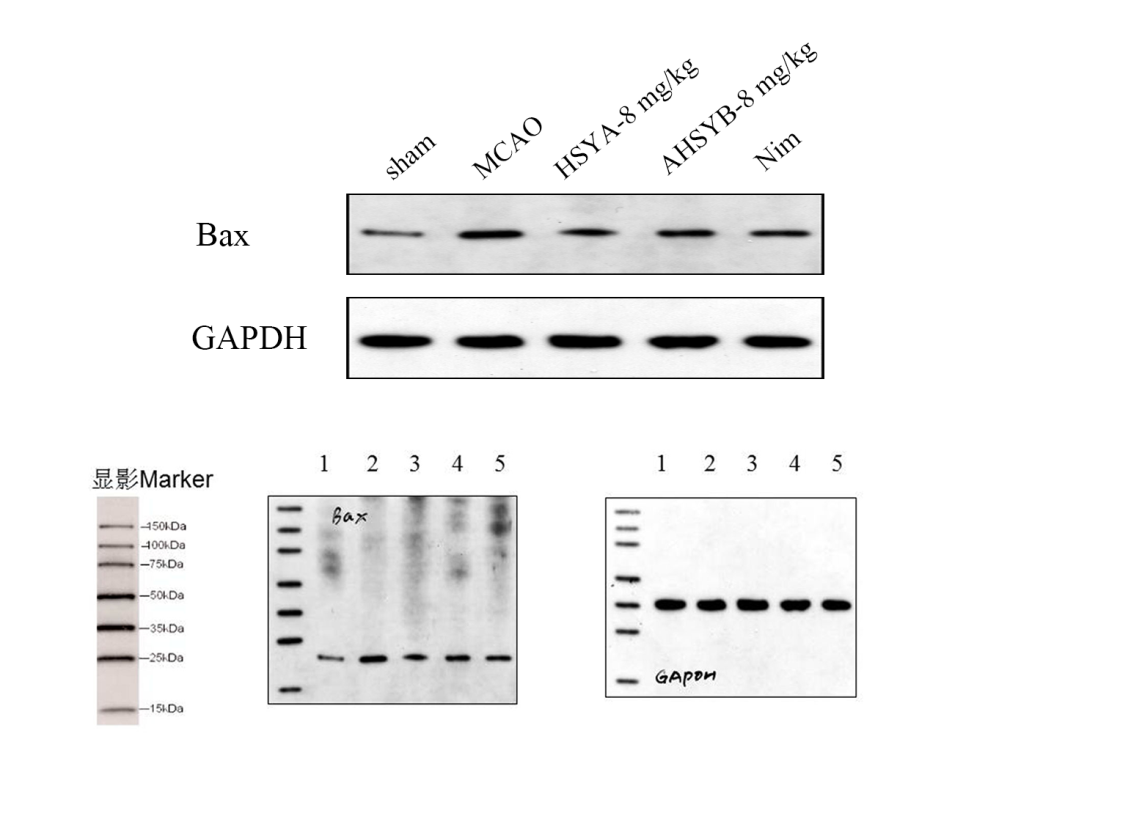


Fig.12


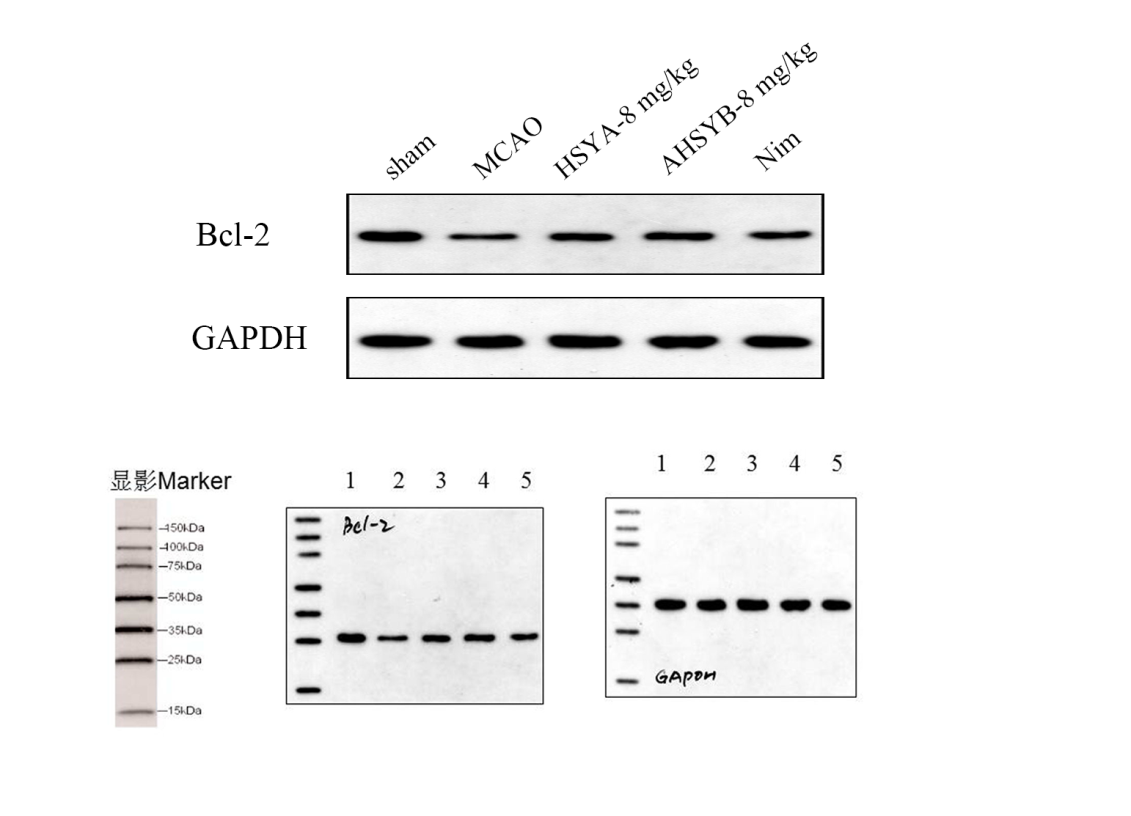


Fig.13


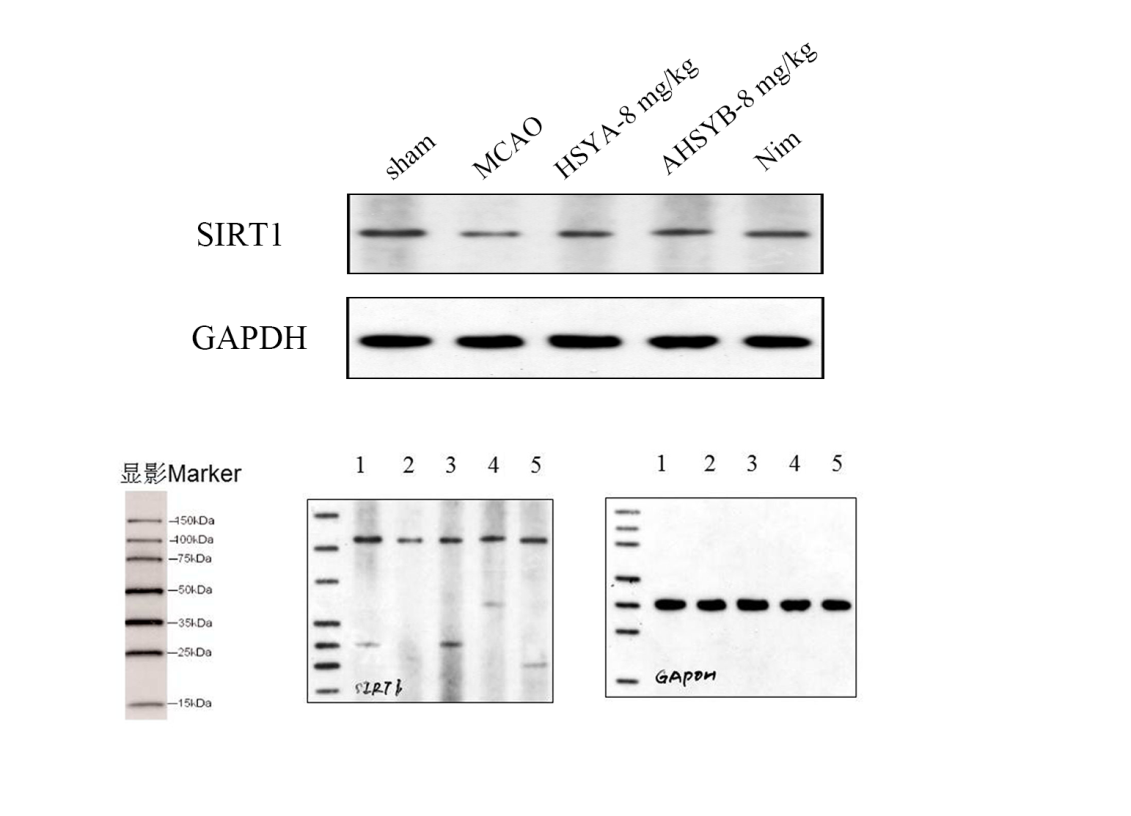


Fig.14


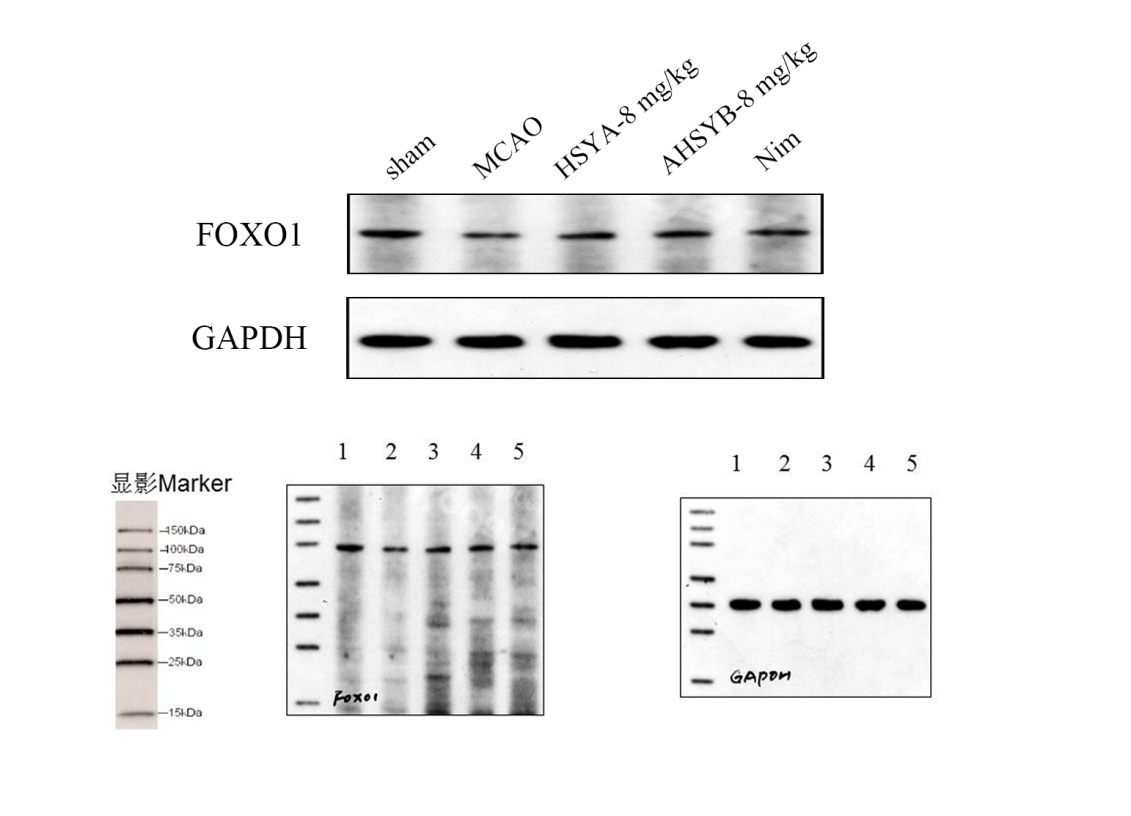


Fig.15


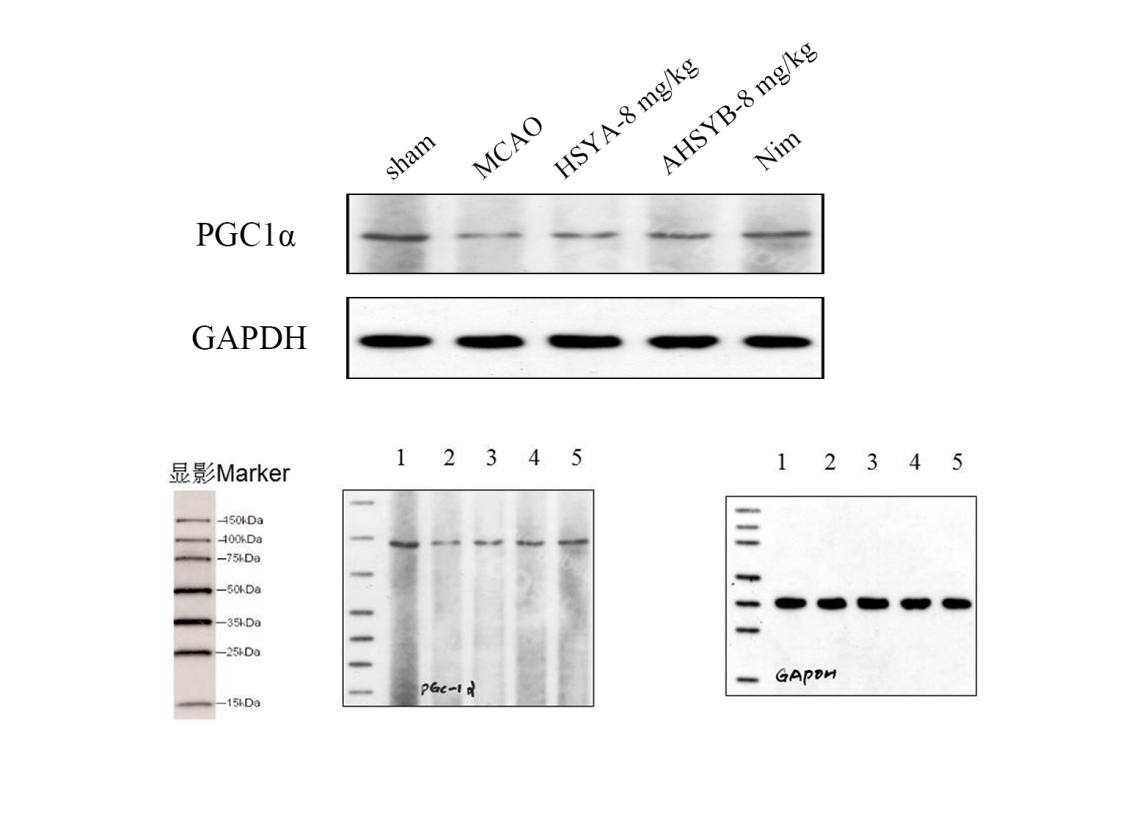

Supplement: Supplementary file 2 [file DataSheet1.DOCX]
