# Supplementary material for: Hydroxysafflor Yellow A and Anhydrosafflor Yellow B Protect Against Cerebral Ischemia/Reperfusion Injury by Attenuating Oxidative Stress and Apoptosis via the Silent Information Regulator 1 Signaling Pathway
Source: Front Pharmacol. 2021 Sep 30;12:739864. doi: 10.3389/fphar.2021.739864 (PMC8514692; doi:10.3389/fphar.2021.739864)
Supplement: Supplementary file 3 [file Image1.PDF]

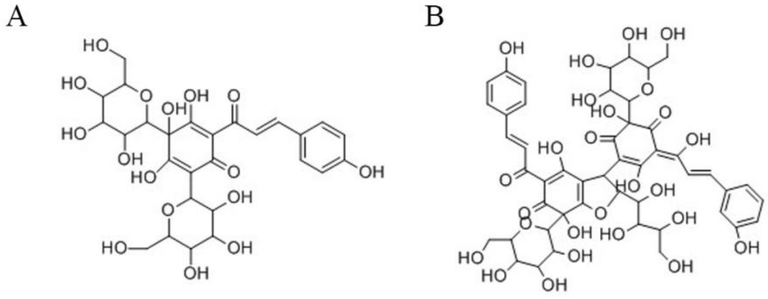

Supplementary figure 1 The chemical structures of HSYA (A) and AHSYB (B).

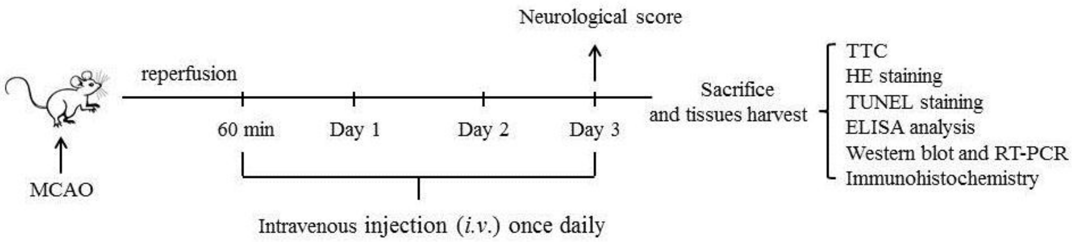

Supplementary figure 2 Administration route of animal experiments.

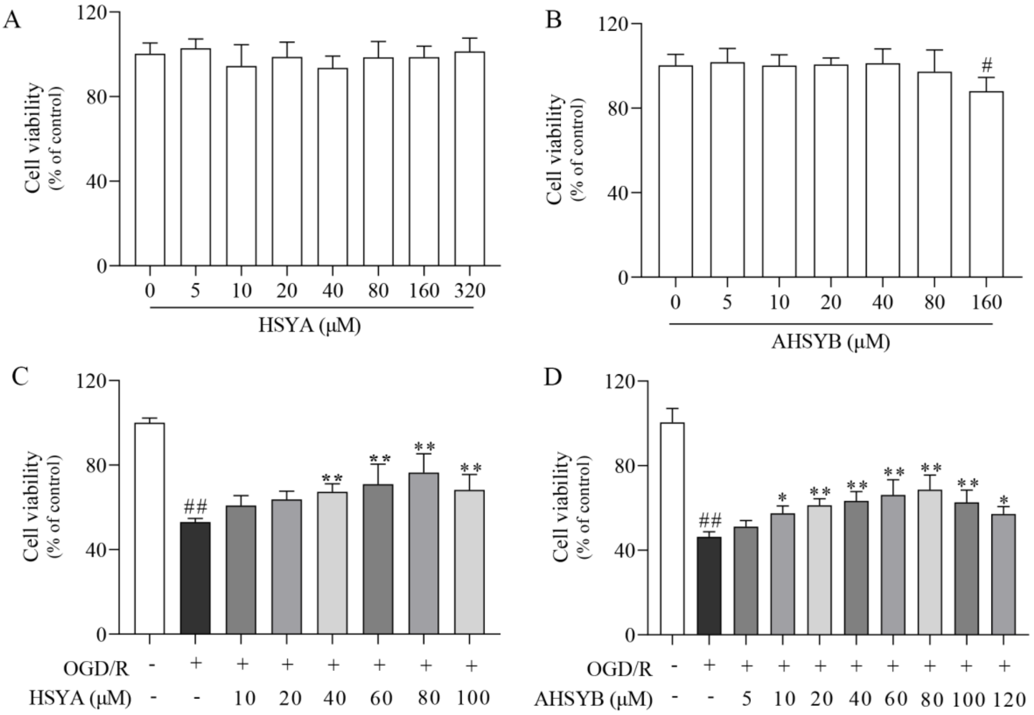

Supplementary figure 3 Determination of suitable concentrations of HSYA and AHSYB in OGD/R-induced hippocampal cells. (A, B) Different concentrations of HSYA and AHSYB treated with cells under normal condition. (C, D) Viability of hippocampal cells treated with HSYA and AHSYB after exposed to OGD/R injury.

Cell viability was measured by CCK-8 assay. Data were expressed as mean ± SD, n=6. $^{\#}p<0.05$, $^{\#\#}p<0.01$ vs. control group; $^{*}p<0.05$, $^{**}p<0.01$ vs. OGD/R group.

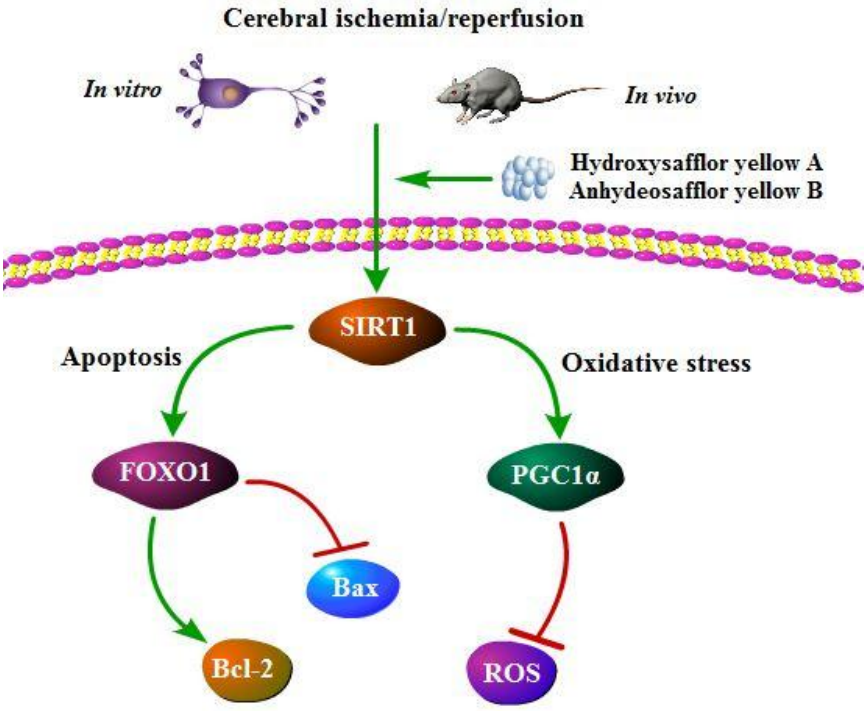

Supplementary figure 4 The mechanism schemes of hydroxysafflor yellow A and anhydeosafflor yellow B on cerebral I/R injury. Hydroxysafflor yellow A and anhydeosafflor yellow B treatment attenuates cerebral I/R injury by alleviating oxidative stress and apoptosis via the SIRT1 signaling pathway.
